# Supplementary material for: Vancomycin-resistant enterococci utilise antibiotic-enriched nutrients for intestinal colonisation
Source: Nat Commun. 2025 Jul 10;16:6376. doi: 10.1038/s41467-025-61731-z (PMC12246219; doi:10.1038/s41467-025-61731-z)
Supplement: Supplementary file 1 — Supplementary Information [file 41467_2025_61731_MOESM1_ESM.pdf]

SUPPLEMENTARY INFORMATION

SUPPLEMENTARY TABLES

Table S1: VRE strains were resistant to broad-spectrum antibiotics that promote VRE intestinal colonisation. The broth microdilution technique was used to measure the MICs of *E. faecalis* and *E. faecium* for vancomycin (VAN), metronidazole (MTZ), ceftriaxone (CRO), piperacillin/tazobactam (TZP) and clindamycin (CLI). Assays were performed under aerobic conditions in Mueller-Hinton broth following EUCAST standards (n = 3).

Strain	VAN (mg/L)	MTZ (mg/L)	CRO (mg/L)	TZP (mg/L)	CLI (mg/L)
E. faecalis (NCTC 12201)	> 128 (resistant)	32 (resistant)	> 128 (resistant)	8 (resistant)	> 128 (resistant)
E. faecium (NCTC 12202)	8 (resistant)	32 (resistant)	8 (resistant)	64 (resistant)	> 128 (resistant)

Table S2: Comparison of *E. faecium* (NCTC 12202) or *E. faecalis* (NCTC 12201) growth in faecal cultures treated with different antibiotics (data from Figs. 1c and 1d). Mixed effects model (one-way) of log transformed CFU/ml with Tukey's multiple comparisons test. * = P ≤ 0.05, ** = P ≤ 0.01, *** = P ≤ 0.001, **** = P ≤ 0.0001. MTZ = metronidazole, CLI = clindamycin, VAN = vancomycin, CRO = ceftriaxone.

Comparison	*E. faecium*		*E. faecalis*	
	Adjusted P value	Summary	Adjusted P value	Summary
Faeces + MTZ vs. Faeces + CLI	0.0858	ns	0.0008	***
Faeces + MTZ vs. Faeces + VAN	0.0129	*	<0.0001	****
Faeces + MTZ vs. Faeces + CRO	<0.0001	****	<0.0001	****
Faeces + CLI vs. Faeces + VAN	0.6969	ns	0.4509	ns
Faeces + CLI vs. Faeces + CRO	0.0008	***	<0.0001	****
Faeces + VAN vs. Faeces + CRO	0.0038	**	0.0009	***

Table S3: Half maximal inhibitory concentrations (IC_{50}) for microbial metabolites and VRE growth. IC_{50} values indicate the concentration of the metabolite that is required to inhibit 50% of vancomycin-resistant *E. faecium* or *E. faecalis* growth. N/A = not available or not applicable.

Metabolite	*E. faecium*			*E. faecalis*		
	NCTC 12202	NCTC 12204	DSM 25698	NCTC 12201	NCTC 13779	DSM 116626
Formate	49.2	47.5	27.8	117.8	36.9	41.5
Acetate	188.7	129.3	77.6	266.9	143.5	131.8
Propionate	66.7	49.6	26.5	36.4	20.7	40.2
Butyrate	48.0	65.0	31.7	80.0	38.4	69.4
Valerate	13.9	23.8	7.7	19.1	18.4	31.7
Isobutyrate	155.5	67.1	32.4	160.4	21.1	132.8
Isovalerate	50.7	19.4	15.5	56.9	17.5	36.8
Lactate	138.8	18.3	119.5	NA	134.6	NA
5-aminovalerate	> 512	N/A	N/A	N/A	N/A	N/A
Ethanol	N/A	119.3	> 512	N/A	134.7	N/A

Table S4: Reagents and oligonucleotides used in this study.

Reagent	Company	Code
3-Trimethylsilylpropionic-2,2,3,3-d_4 acid sodium salt	Merck	269913-1G
5-aminovalerate	Merck	123188-5G
Acetic acid	Merck	A6283-100ML
Adenosine	TCI Europe	A0152-5G
Agar	VWR	84609.05
Ammonium sulfate	Sigma	A4418-500G
Arabinogalactan	TCI Europe	A1328-100G
Bile salts	Fisher Scientific	10128872
Boric acid	Sigma	B6768-1KG
Brain heart infusion broth	Sigma	53286-500G
Brilliance CRE agar	Fisher Scientific	13205349
Brilliance VRE agar	Fisher Scientific	11924112
Butyric acid	Merck	B103500-100ML
Calcium chloride anhydrous	Fisher Scientific	10515671
Casein	VWR	A13707.36
Casein hydrolysate	Merck	22090-100G
Cobalt (II) chloride	Sigma	C8661-25G
Columbia Agar	VWR	84621.05
Copper (II) chloride	Sigma	C3279-100G
Defibrinated horse blood	SLS	MED1306
Deuterium oxide	Goss Scientific	DLM-4-100G
D-Fructose	Merck	F0127-100G
D-Galactose	Fluorochem	218380-25G
D-Glucose	VWR	101174Y
D-Maltose	TCI Europe	M0037-25G
D-Mannose	TCI Europe	M0037-25G
DNeasy PowerLyzer PowerSoil Kit	Qiagen	12855-100
D-Ribose	Fluorochem	078886-25G
D-Trehalose dihydrate	Fluorochem	243212-25G
D-Xylose	Fluorochem	209047-100G
Escherichia coli DNA	Merck	D4889-1UN
Ethanol	Fisher Scientific	10437341
Ethylenediaminetetraacetic acid	Sigma	E5134-50G
Fastidious anaerobic agar	SLS	MED1332
Formic acid	VWR	20320.295
Gifu anaerobic broth	VWR	HIMEM1801-500G
Guanosine	TCI Europe	G0171-5G
Hemin	Merck	51280-1G
Hydrochloric acid	VWR	20252.29
Inulin	VWR	A18425.18
Iron sulphate	Sigma	7782-63-0
Isobutyric acid	Merck	I1754-100ML
Isovaleric acid	Merck	129542-100ML

Reagent	Company	Code
Kao and Michayluk vitamin solution	Merck	K3129
L-Alanine	TCI Europe	A0179-25G
L-Arabinose	Fluorochem	078969-25G
L-Arginine hydrochloride	TCI Europe	A0528-25G
L-Aspartic acid	TCI Europe	A0546-25G
L-Cysteine hydrochloride monohydrate	Merck	C7880-100G
L-Fucose	Fluorochem	216288-5G
L-Glutamic acid	TCI Europe	G0059-25G
L-Glycine	TCI Europe	G0099-25G
L-Isoleucine	TCI Europe	I0181-25G
L-Leucine	TCI Europe	L0029-25G
L-Lysine hydrochloride	TCI Europe	L0071-25G
L-Methionine	TCI Europe	M0099-25G
L-Phenylalanine	TCI Europe	P0134-25G
L-Proline	TCI Europe	P0481-25G
L-Threonine	TCI Europe	T0230-25G
L-Tryptophan	TCI Europe	T0541-25G
L-Tyrosine	TCI Europe	T0550-25G
L-Valine	TCI Europe	V0014-25G
Magnesium chloride	Sigma	7786-30-3
Magnesium sulphate heptahydrate	Fisher Scientific	10424511
Manganese (II) chloride	Sigma	M5005-100G
Menadione	Merck	M9429-25G
MiSeq Reagent Kit V3	Illumina	MS-102-3003
Mucin from porcine stomach type II	Merck	M2378-500G
Muller-Hinton broth	Merck	70192-100G
N-acetylglucosamine	TCI Europe	A0092-25G
NEBNext Library Quant Kit for Illumina	New England Biolabs	E7630L
Nickel dichloride	Sigma	654507-5G
Nicotinic acid	Sigma	72309-100G
Pantethine	Sigma	P2126-1G
PCR grade water	Merck	3315932001
Pectin citrus	VWR	J61021.36
Peptone water	Merck	70179-500G
Platinum Supermix with ROX	Life Technologies	11730017
Potassium phosphate dibasic	Merck	P3786-500G
Potassium phosphate monobasic	Merck	P5655-500G
Propionic acid	Merck	P1386-500ML
Proteose peptone	Merck	82450-100G
RMPI-1640 amino acid solution	Merck	R7131
SequalPrep Normalisation Plate Kit	Life Technologies	A1051001
Sodium azide	Merck	71289-5G
Sodium bicarbonate	Merck	S6014-500G
Sodium chloride	Merck	S7653-1KG
Sodium DL-lactate	Merck	71720-5G

Reagent	Company	Code
Sodium fumerate	Fluorochem	F862900-25G
Sodium hydroxide	Fisher Scientific	10478030
Sodium phosphate dibasic	Merck	71640-250G
Sodium phosphate monobasic	Fisher Scientific	389872500
Sodium pyruvate	SLS	P2256-5G
Sodium succinate dibasic hexahydrate	SLS	S2378-100G
Soluble starch	SLS	CHE3622
Starch	Merck	S5127-5KG
Sucrose	Merck	84100-250G
Thymine	TCI Europe	T0234-5G
Tri-sodium citrate monobasic	VWR	DIUKTR19204-535
Tryptic Soy Broth	Sigma	22092-500G
Tween 80	Sigma	P1754-25ML
Uracil	TCI Europe	U0013-25G
Valeric acid	Merck	240370-100ML
Xylan	Universal Biologicals	38500.02
Yeast extract	Merck	70161-500G
Zinc chloride	Sigma	Z0152-100G
16S sequencing primer: 28F-YM (forward primer, MiSeq adapter sequences in bold)	Eurofins	5'-**TCGTCGGCAGCGTCAGATGTGTATAAGAGACAG**GAGTTTGATYMTGGCTCAG-3'
16S sequencing primer: 28F-Borrellia (forward primer, MiSeq adapter sequences in bold)	Eurofins	5'-**TCGTCGGCAGCGTCAGATGTGTATAAGAGACAG**GAGTTTGATCCTGGCTTAG-3'
16S sequencing primer: 28F-Chloroflex (forward primer, MiSeq adapter sequences in bold)	Eurofins	5'-**TCGTCGGCAGCGTCAGATGTGTATAAGAGACAG**GAATTTGATCTTGGTTCAG-3'
16S sequencing primer: 28F-Bifdo (forward primer, MiSeq adapter sequences in bold)	Eurofins	5'-**TCGTCGGCAGCGTCAGATGTGTATAAGAGACAG**GGGTTCGATTCTGGCTCAG-3'
16S sequencing primer: 388R (reverse primer, MiSeq adapter sequences in bold)	Eurofins	5'-**GTCTCGTGGGCTCGGAGATGTGTATAAGAGACAG**TGCTGCCTCCCGTAGGAGT-3'
16S rRNA gene qPCR: BactQUANT forward primer	Eurofins	5′-CCTACGGGAGGCAGCA-3′
16S rRNA gene qPCR: BactQUANT reverse primer	Eurofins	5′-GGACTACCGGGTATCTAATC-3′
16S rRNA gene qPCR: BactQUANT probe	Life Technologies	4316034, [6FAM] 5′-CAGCAGCCGCGGTA-3′ [MGBNFQ]

Table S5: Concentrations of microbial metabolites tested in the metabolite inhibition assays. Concentrations were based on measurements taken from faecal samples from 12 healthy human donors from Yip *et al*. (2023)[1].

Metabolite	Low concentration (mM)	Average concentration (mM)	High concentration (mM)
Formate	0.05	0.10	0.15
Acetate	10	65	120
Propionate	5	15	35
Butyrate	3	15	40
Valerate	0.5	3.5	12
Isobutyrate	0.5	2	5
Isovalerate	0.5	2	5
Lactate	0.3	0.7	1.2
5-aminovalerate	0.3	1	4
Ethanol	0.6	10	60

SUPPLEMENTARY FIGURES

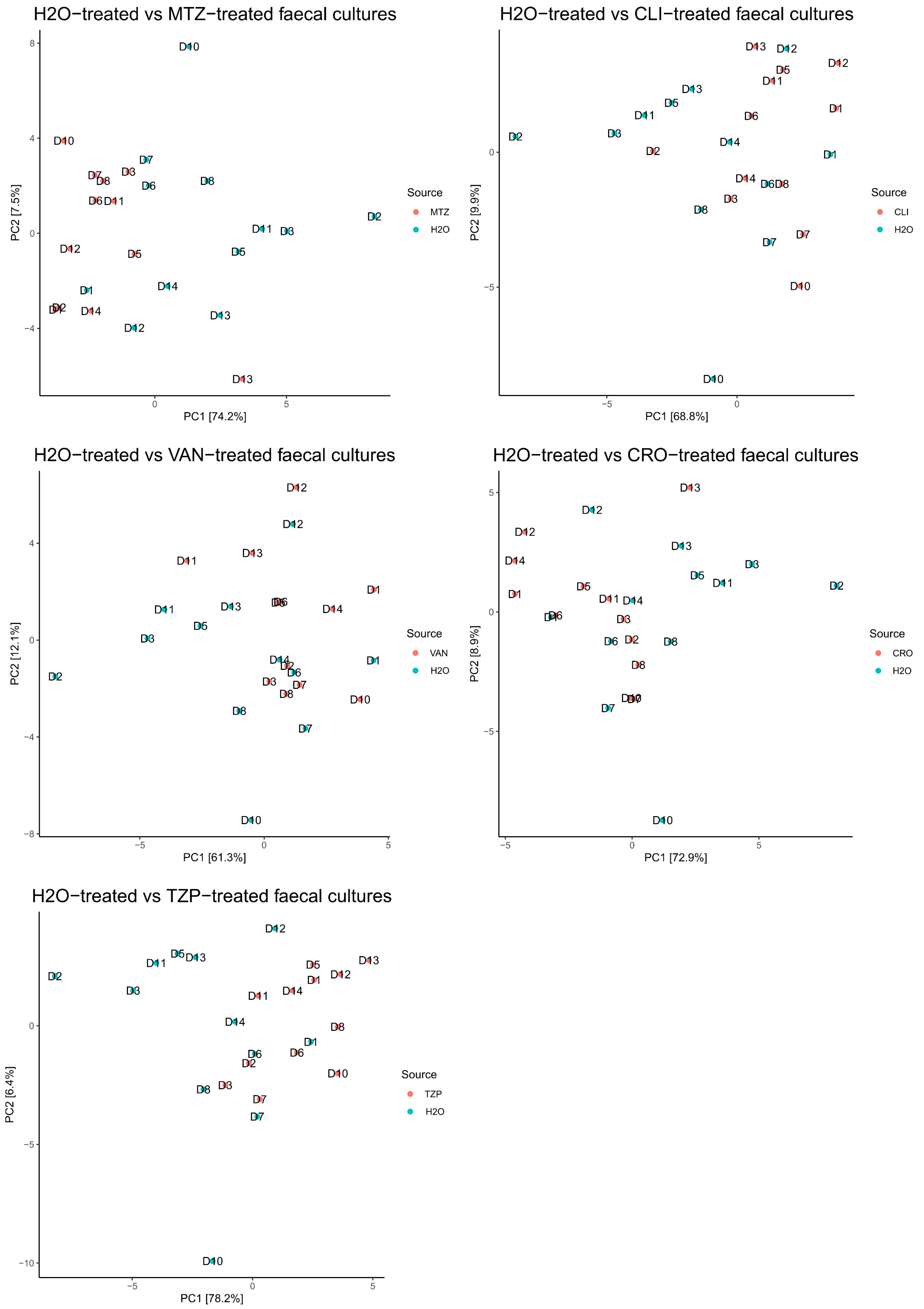

Figure S1: Principal component analysis (PCA) plots of water-treated vs antibiotic-treated faecal culture samples. Plots used log-transformed absolute abundance data at the family level. MTZ = metronidazole, CLI = clindamycin, VAN = vancomycin, CRO = ceftriaxone, TZP = piperacillin/tazobactam, H_2O = water, PC1 = principal component 1, PC2 = principal component 2. D1 - D12 = Donor 1 – Donor 12.

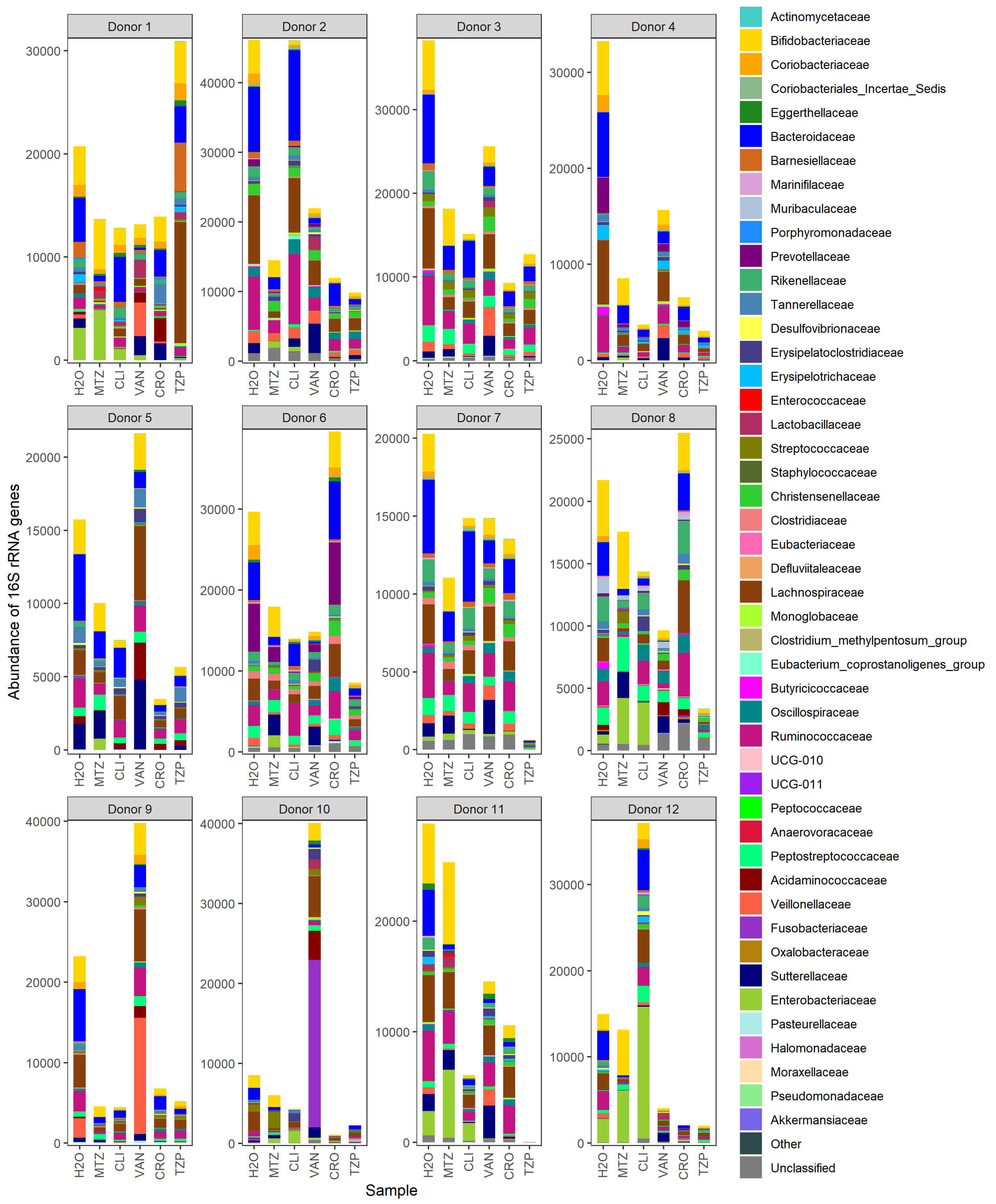

Figure S2: Stacked bar plots showing the absolute abundances of bacterial families found in water-treated and antibiotic-treated faecal cultures, presented by individual donors. MTZ = metronidazole, CLI = clindamycin, VAN = vancomycin, CRO = ceftriaxone, TZP = piperacillin/tazobactam, H_2O = water.

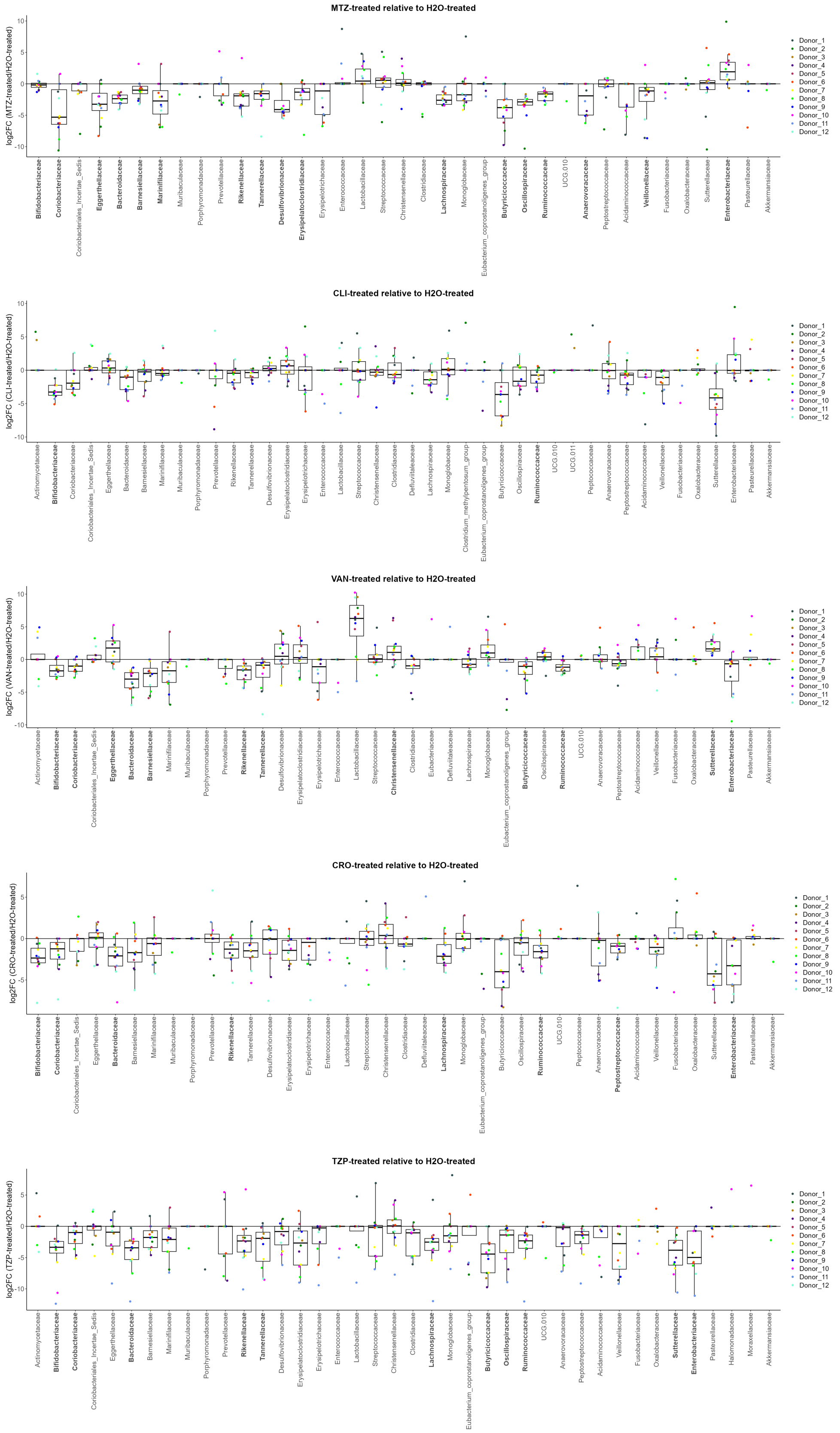

Figure S3: Log2FC of bacterial families in antibiotic-treated faecal cultures as compared to water-treated faecal cultures by individual donor. Bacterial families shown in bold font on the x-axis had a significant change in antibiotic-treated faecal cultures as compared to water-treated faecal cultures. n = 12 healthy human faecal donors, Wilcoxon signed rank test (two-sided) of log-transformed abundances with Benjamini Hochberg false discovery rate (FDR) correction, $p < 0.05$. The box shows the median as a line in the box, the lower and upper hinges correspond to the first and third quartiles, and the whiskers extend to the maximum and minimum values no further than 1.5*IQR from the hinge. MTZ = metronidazole, CLI = clindamycin, VAN = vancomycin, CRO = ceftriaxone, TZP = piperacillin/tazobactam, H_2O = water.

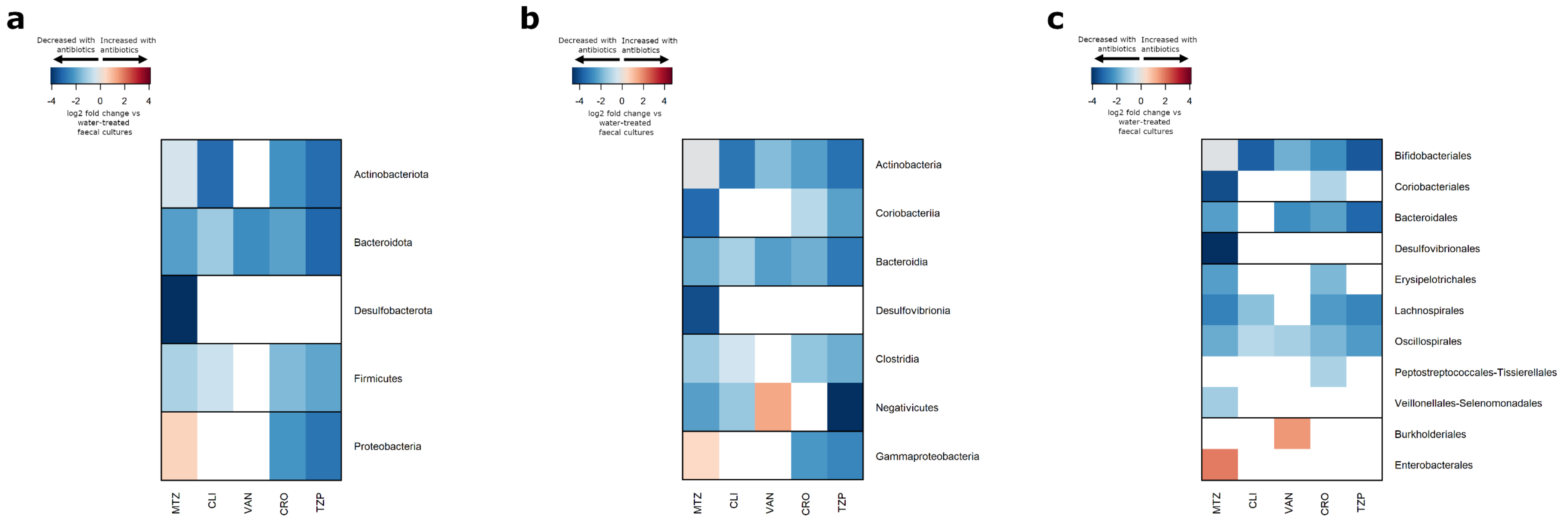

Figure S4: Antibiotic treatment significantly reduced the abundance of gut commensals at the phylum, class, and order levels. Heatmaps illustrating the log2 fold change (antibiotic-treated relative to water-treated) in bacterial taxa that were significantly decreased (shown in blue) or increased (shown in red) in faecal cultures following treatment with antibiotics that promote VRE intestinal colonisation. Taxa that were not significantly changed with antibiotic treatment were not plotted (shown in white). Changes are plotted at (a) phylum level, (b) class level, and (c) order level. n = 12 healthy human faecal donors, Wilcoxon signed rank test (two-sided) of log-transformed abundances with Benjamini Hochberg false discovery rate (FDR) correction, $p < 0.05$. MTZ = metronidazole, CLI = clindamycin, VAN = vancomycin, CRO = ceftriaxone, TZP = piperacillin/tazobactam.

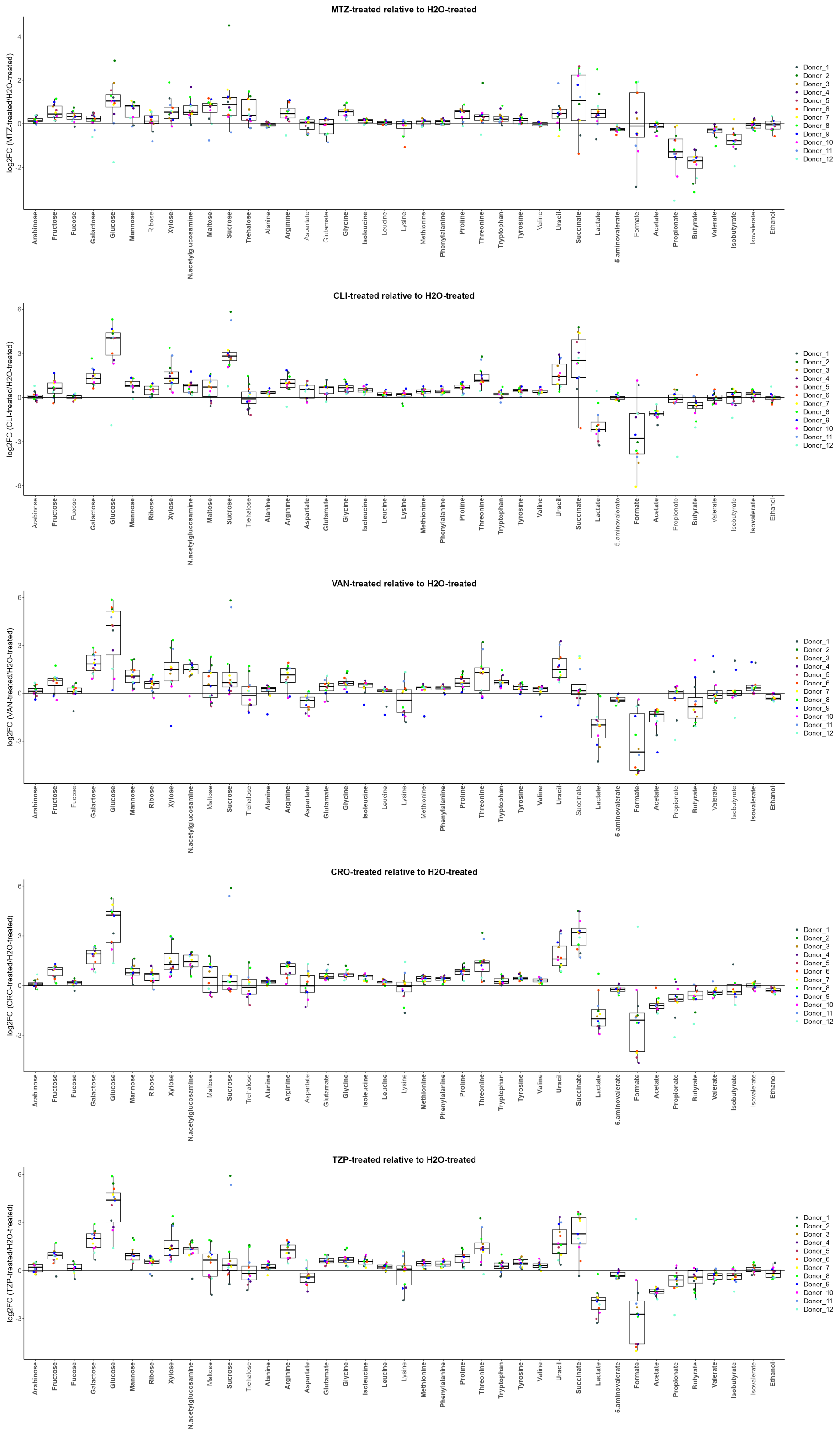

Figure S5: Log2FC of nutrients and metabolites in antibiotic-treated faecal cultures as compared to water-treated faecal cultures by individual donor. Nutrients or metabolites shown in bold font on the x-axis had a significant change in antibiotic-treated faecal cultures as compared to water-treated faecal cultures. n = 12 healthy human faecal donors, Wilcoxon signed rank test (two-sided) of log-transformed abundances with Benjamini Hochberg false discovery rate (FDR) correction, $p < 0.05$. The box shows the median as a line in the box, the lower and upper hinges correspond to the first and third quartiles, and the whiskers extend to the maximum and minimum values no further than 1.5*IQR from the hinge. MTZ = metronidazole, CLI = clindamycin, VAN = vancomycin, CRO = ceftriaxone, TZP = piperacillin/tazobactam, H_2O = water.

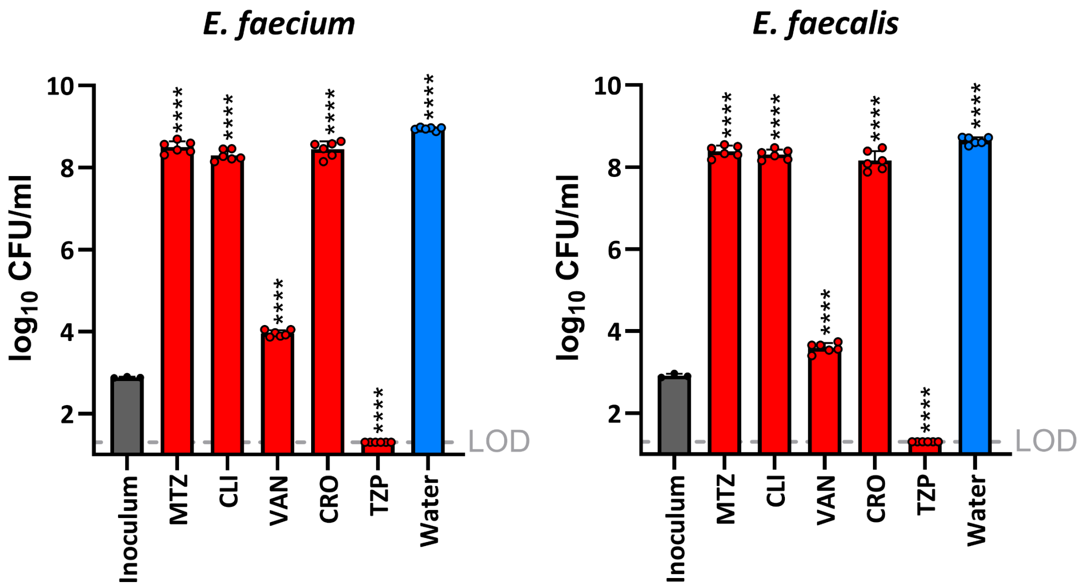

Figure S6: VRE strains can grow in faecal concentrations of MTZ, CLI, VAN and CRO but are killed by faecal concentrations of TZP. Vancomycin-resistant *E. faecium* (NCTC 12202) and *E. faecalis* (NCTC 12201) were grown anaerobically in gut growth medium supplemented with water or with one of the following antibiotics at concentrations mimicking those measured in human faeces: metronidazole (MTZ), clindamycin (CLI), vancomycin (VAN), ceftriaxone (CRO), or piperacillin/tazobactam (TZP). Antibiotic-treated samples (shown in red) or water-treated samples (shown in blue) were compared to the inoculum (shown in grey) using a one-way ANOVA with Dunnett's multiple comparison test. n=6 replicates from 3 independent experiments for the antibiotic- or water-treated groups, n=3 replicates from 3 independent experiments from the inoculum group, with data shown as mean ± SD. LOD = limit of detection.

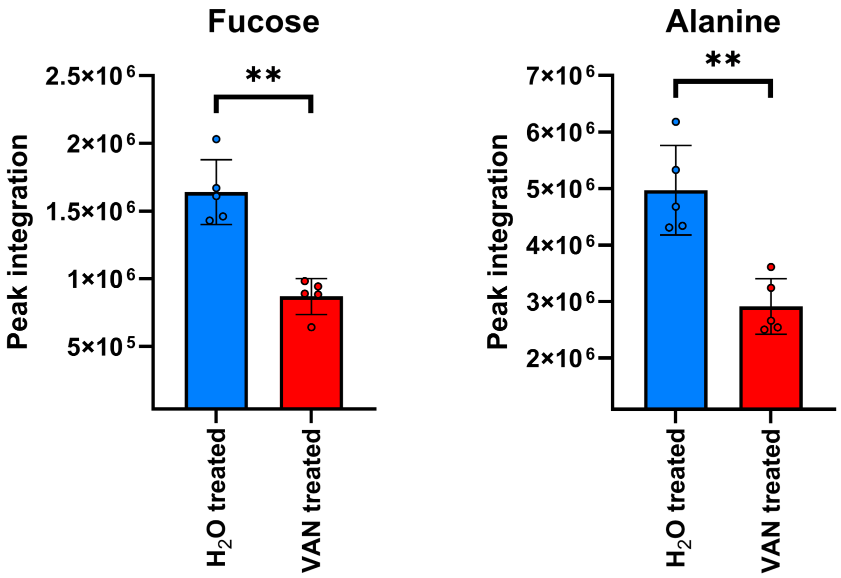

Figure S7: Nutrients decreased in mouse faeces with vancomycin treatment. Comparison of nutrients measured in VAN-treated mouse faeces (shown in red) to H_2O-treated mouse faeces (shown in blue) used an unpaired t-test (two-sided) with Benjamini Hochberg FDR. n = 5 mice per group from one independent experiment, with data shown as mean ± SD. ** = P ≤ 0.01. VAN = vancomycin, H_2O = water.

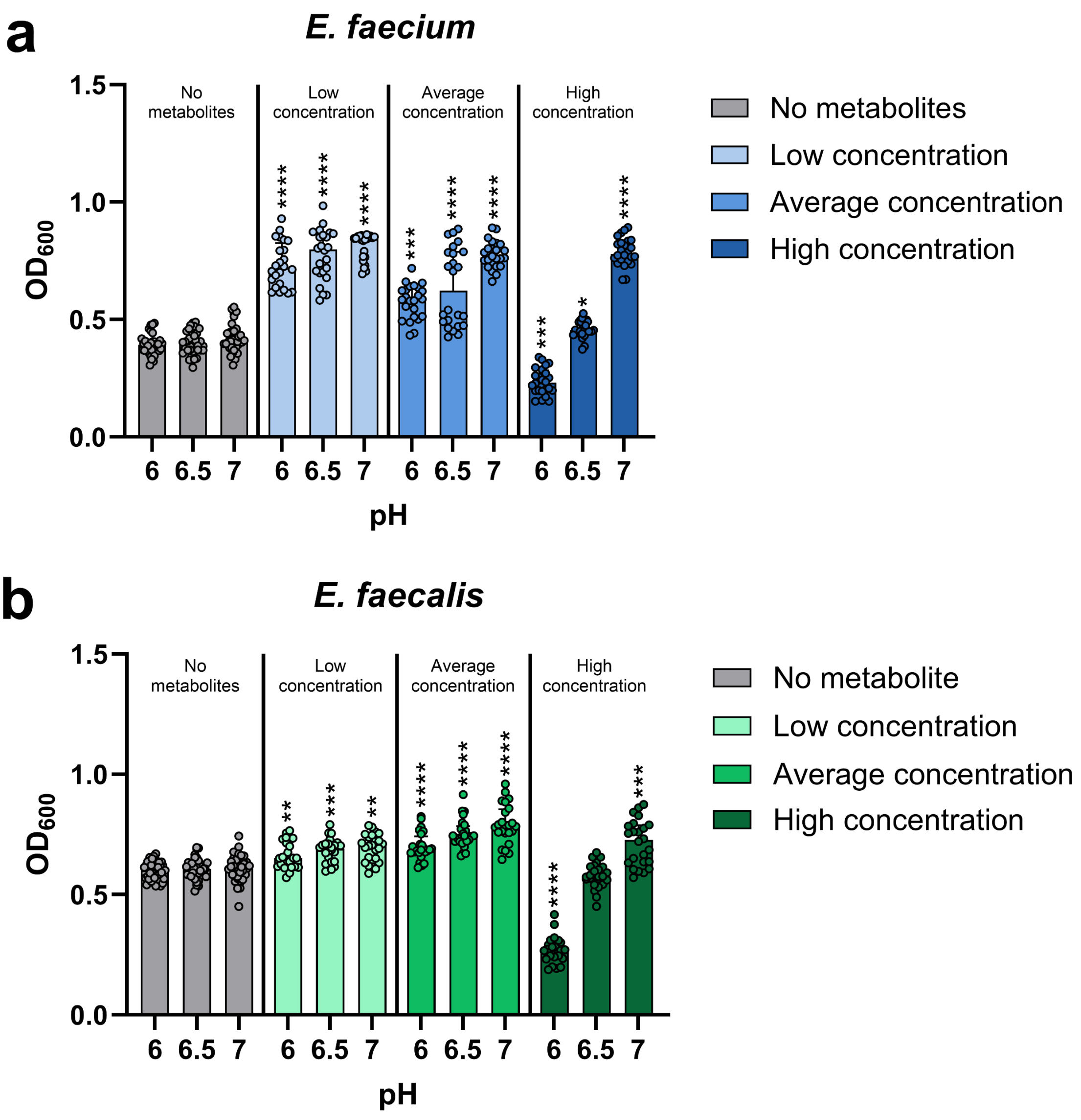

Figure S8: VRE growth was suppressed at pH 6 by a mixture of 10 microbial metabolites at high concentrations. (a) Vancomycin-resistant *E. faecium* (NCTC 12202) and (b) vancomycin-resistant *E. faecalis* (NCTC 12201) were grown in tryptic soy broth supplemented with a mixture of 10 metabolites mimicking the low, average, or high concentrations measured in healthy human faeces, or unsupplemented (No metabolite control). Broth was adjusted to pH 6, 6.5, or 7 to mimic the pH of the healthy large intestine, and cultures were incubated under anaerobic conditions overnight. Data shown as medians ± IQR, with 24-36 replicates from 3 independent experiments. Kruskal-Wallis with Dunn's multiple comparison test comparing the no metabolite control to each concentration of the metabolite mixture at the same pH. * P ≤ 0.05, ** = P ≤ 0.01, *** = P ≤ 0.001, **** = P ≤ 0.0001. OD_{600} = optical density at 600 nm.

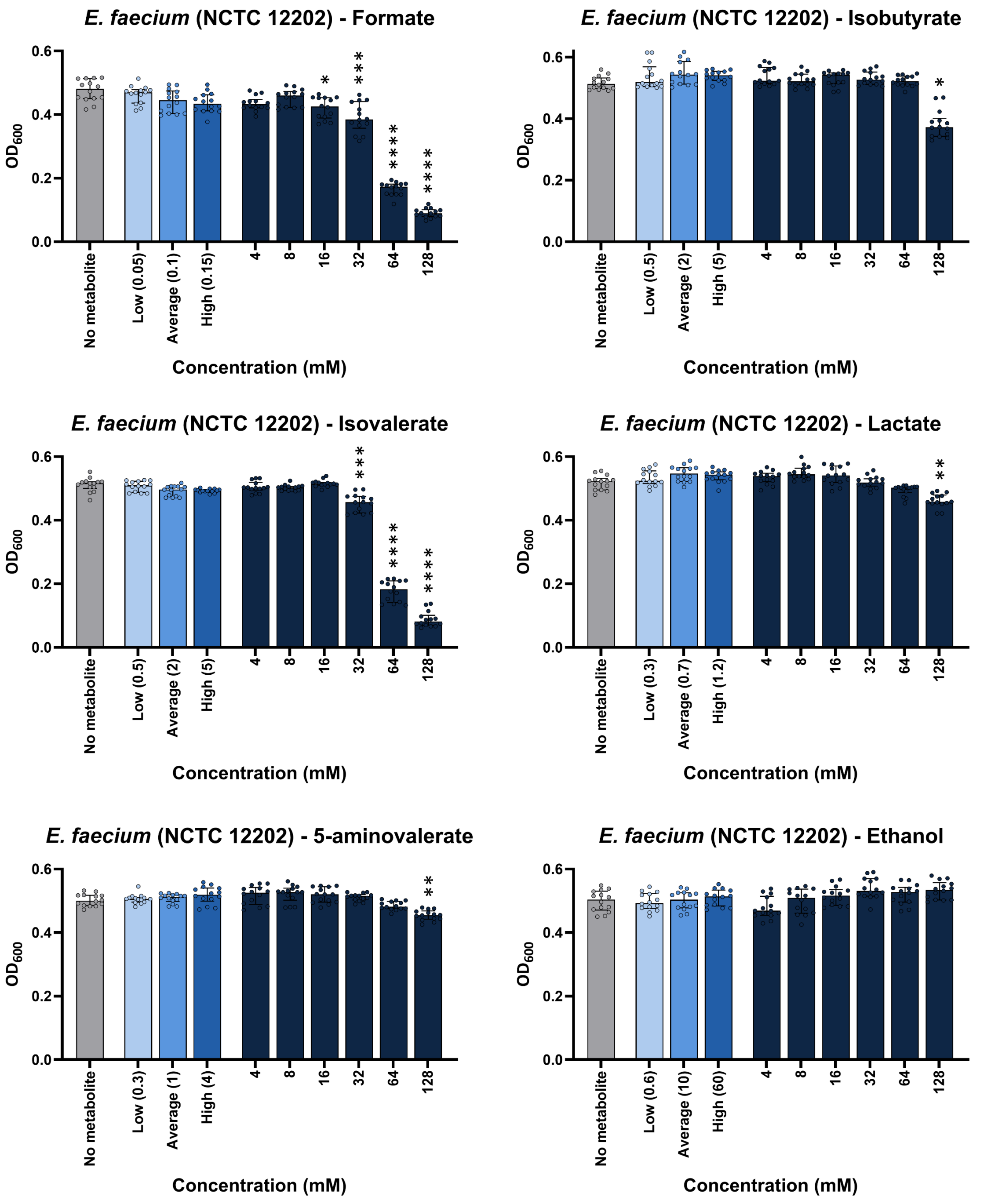

Figure S9: Some individual microbial metabolites did not suppress vancomycin-resistant *E. faecium* (NCTC 12202) growth at physiological concentrations. Vancomycin-resistant *E. faecium* (NCTC 12202) was grown in tryptic soy broth (pH 6) supplemented with an individual metabolite at low, average or high concentrations measured in healthy human faeces, concentrations spanning a defined range (4-128 mM), or unsupplemented (no metabolite control). Cultures were incubated under anaerobic conditions overnight. Growth was measured with 14 replicates in two independent experiments. Kruskal-Wallis test with Dunn's multiple comparison test (each metabolite was compared to the no metabolite control). * = $P \leq 0.05$, ** = $P \leq 0.01$, *** = $P \leq 0.001$, **** = $P \leq 0.0001$. Data are presented as medians ± IQR. Growth of *E. faecium* without metabolites shown in grey. Growth of *E. faecium* with supplemented metabolites shown in shades of blue. Optical density at 600 nm, OD_{600}.

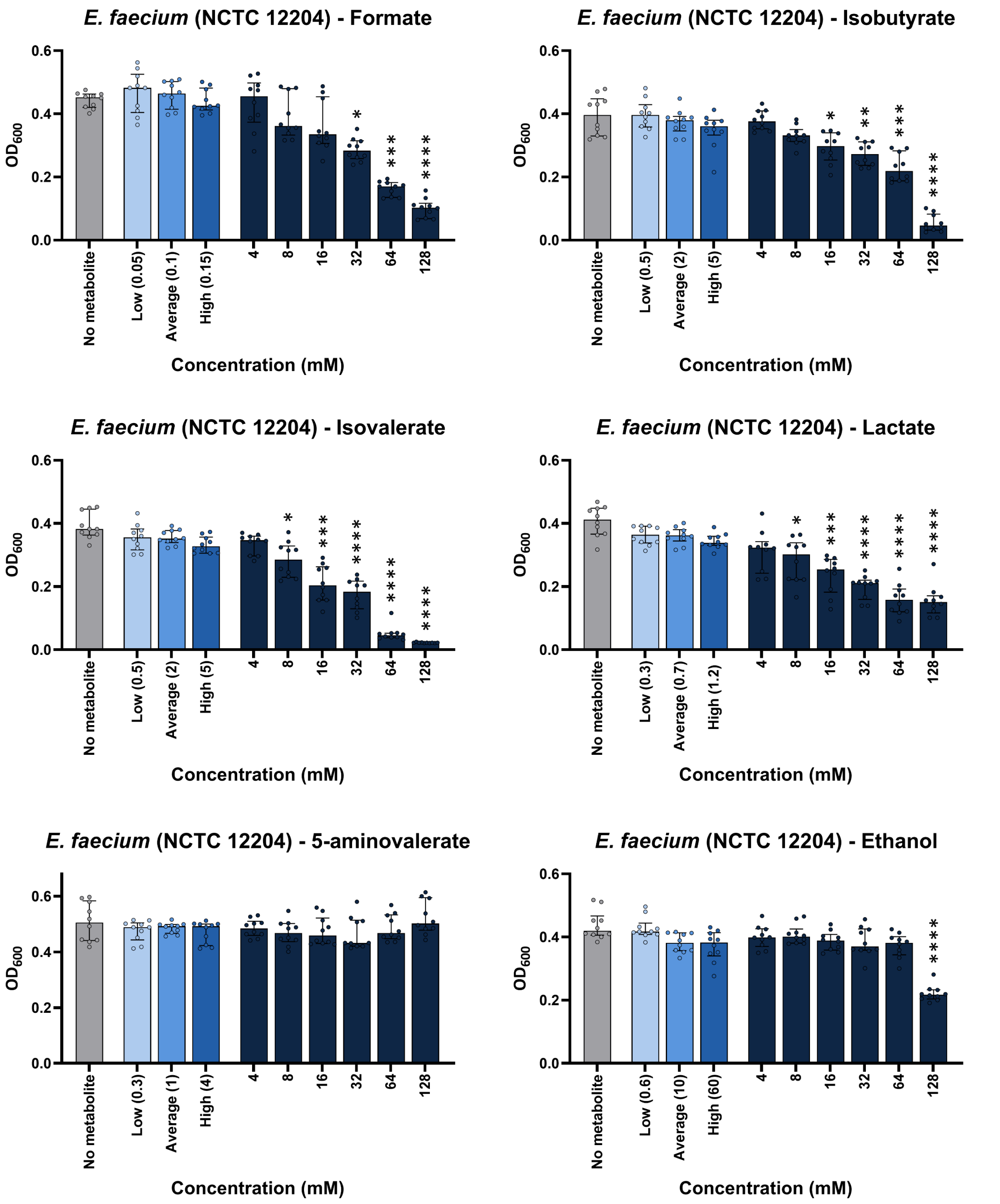

Figure S10: Some individual microbial metabolites did not suppress vancomycin-resistant *E. faecium* (NCTC 12204) growth at physiological concentrations. Vancomycin-resistant *E. faecium* (NCTC 12204) was grown in tryptic soy broth (pH 6) supplemented with an individual metabolite at low, average or high concentrations measured in healthy human faeces, concentrations spanning a defined range (4-128 mM), or unsupplemented (no metabolite control). Cultures were incubated under anaerobic conditions overnight. Growth was measured with 10 replicates in two independent experiments. Kruskal-Wallis test with Dunn's multiple comparison test (each metabolite was compared to the no metabolite control). * = $P \leq 0.05$, ** = $P \leq 0.01$, *** = $P \leq 0.001$, **** = $P \leq 0.0001$. Data are presented as medians ± IQR. Growth of *E. faecium* without metabolites shown in grey. Growth of *E. faecium* with supplemented metabolites shown in shades of blue. Optical density at 600 nm, OD_{600}.

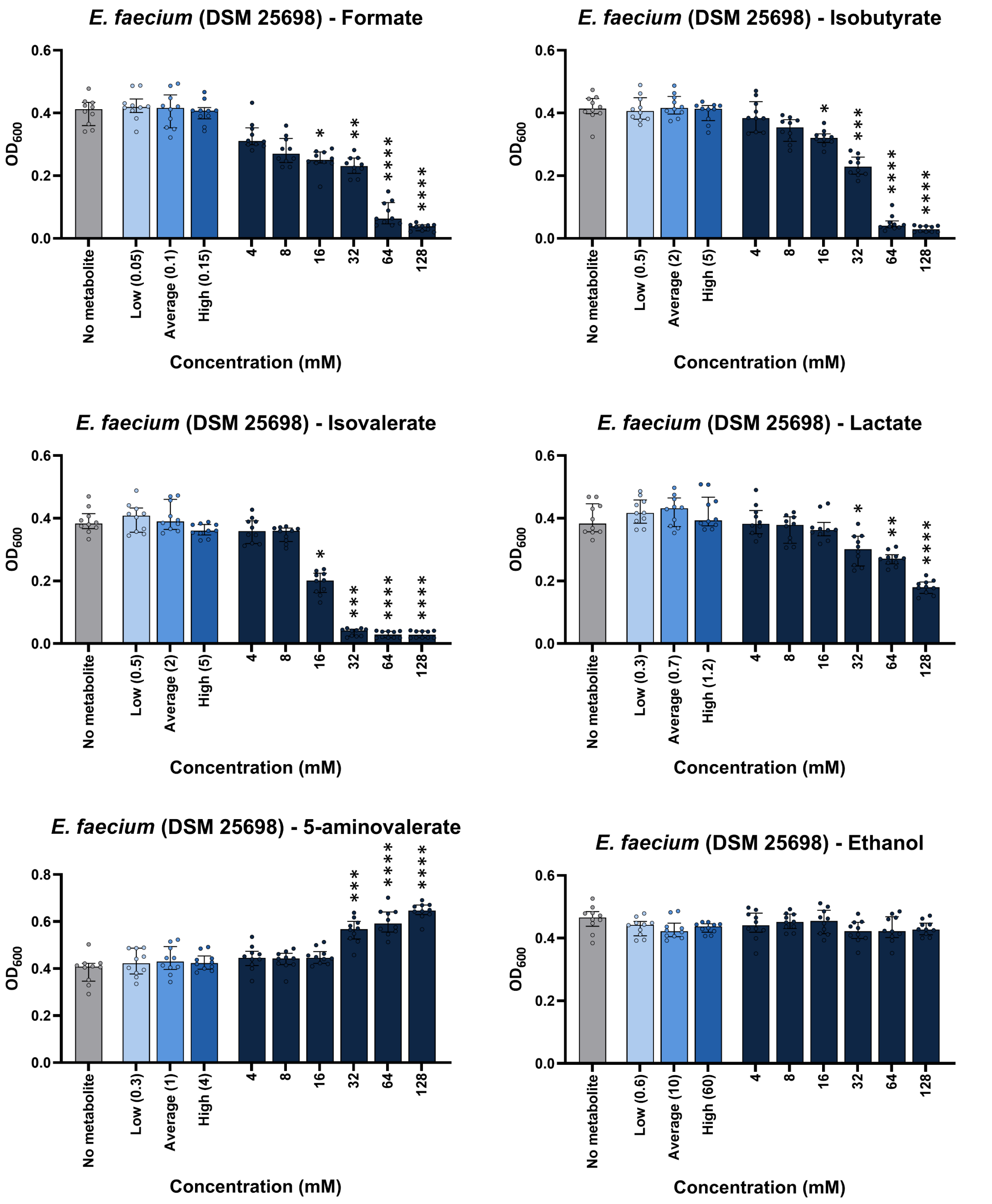

Figure S11: Some individual microbial metabolites did not suppress vancomycin-resistant *E. faecium* (DSM 25698) growth at physiological concentrations. Vancomycin-resistant *E. faecium* (DSM 25698) was grown in tryptic soy broth (pH 6) supplemented with an individual metabolite at low, average or high concentrations measured in healthy human faeces, concentrations spanning a defined range (4-128 mM), or unsupplemented (no metabolite control). Cultures were incubated under anaerobic conditions overnight. Growth was measured with 10 replicates in two independent experiments. Kruskal-Wallis test with Dunn's multiple comparison test (each metabolite was compared to the no metabolite control). * = $P \leq 0.05$, ** = $P \leq 0.01$, *** = $P \leq 0.001$, **** = $P \leq 0.0001$. Data are presented as medians ± IQR. Growth of *E. faecium* without metabolites shown in grey. Growth of *E. faecium* with supplemented metabolites shown in shades of blue. Optical density at 600 nm, OD_{600}.

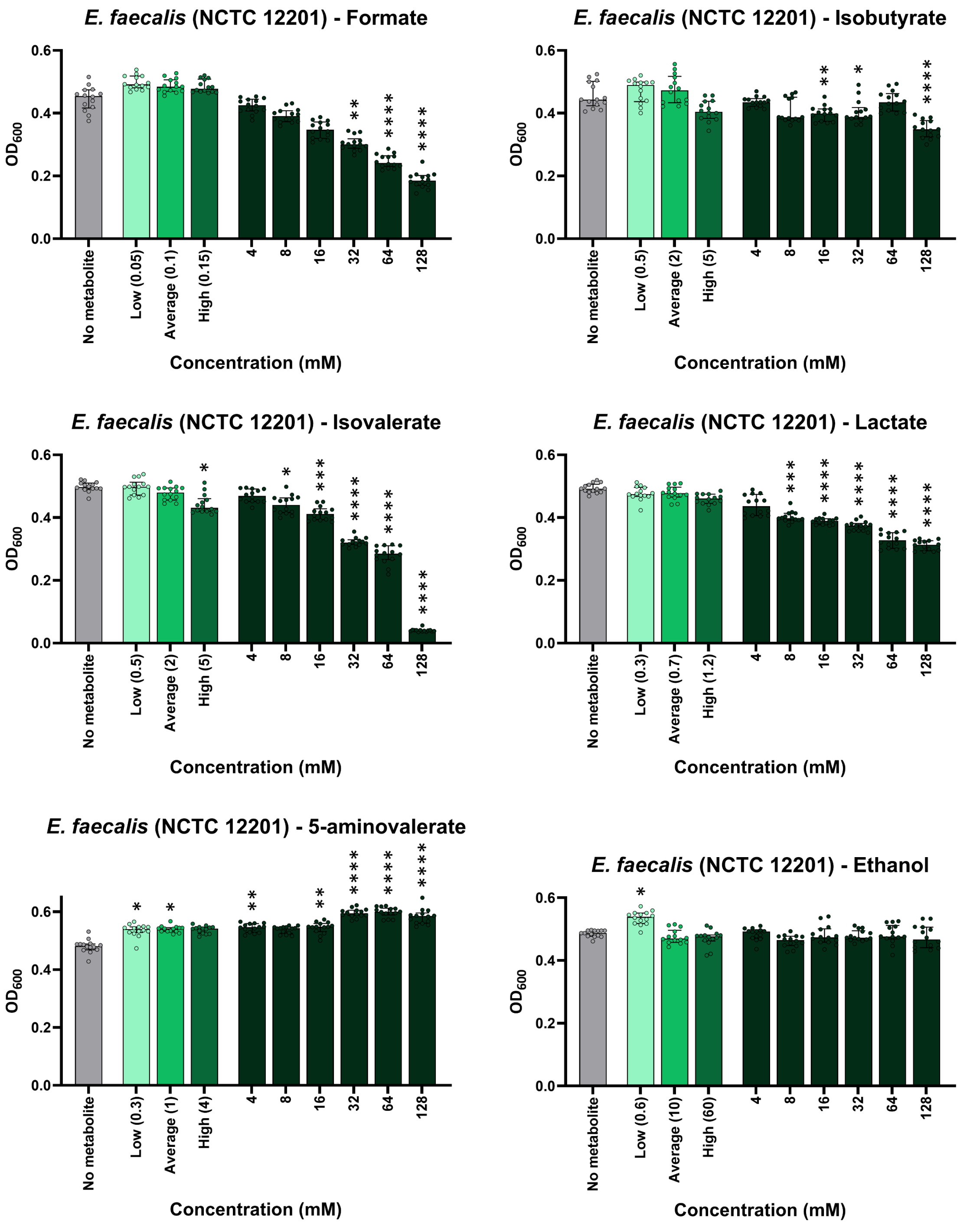

Figure S12: Some individual microbial metabolites did not suppress vancomycin-resistant *E. faecalis* (NCTC 12201) growth at physiological concentrations. Vancomycin-resistant *E. faecalis* (NCTC 12201) was grown in tryptic soy broth (pH 6) supplemented with an individual metabolite at low, average or high concentrations measured in healthy human faeces, concentrations spanning a defined range (4-128 mM), or unsupplemented (no metabolite control). Cultures were incubated under anaerobic conditions overnight. Growth was measured with 14 replicates in two independent experiments. Kruskal-Wallis test with Dunn's multiple comparison test (each metabolite was compared to the no metabolite control). * = $P \leq 0.05$, ** = $P \leq 0.01$, *** = $P \leq 0.001$, **** = $P \leq 0.0001$. Data are presented as medians ± IQR. Growth of *E. faecalis* without metabolites shown in grey. Growth of *E. faecalis* with supplemented metabolites shown in shades of green. Optical density at 600 nm, OD_{600}.

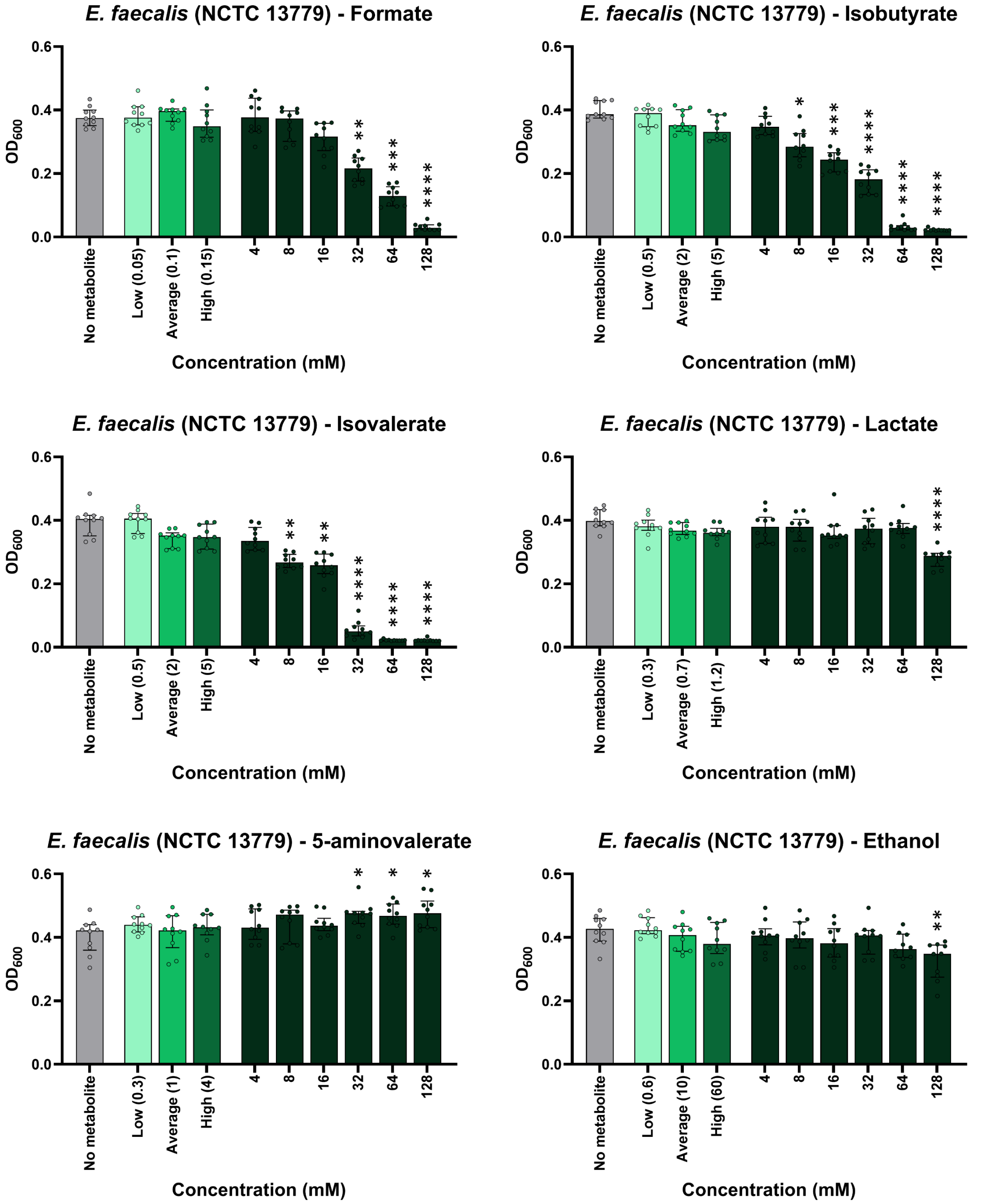

Figure S13: Some individual microbial metabolites did not suppress vancomycin-resistant *E. faecalis* (NCTC 13779) growth at physiological concentrations. Vancomycin-resistant *E. faecalis* (NCTC 13779) was grown in tryptic soy broth (pH 6) supplemented with an individual metabolite at low, average or high concentrations measured in healthy human faeces, concentrations spanning a defined range (4-128 mM), or unsupplemented (no metabolite control). Cultures were incubated under anaerobic conditions overnight. Growth was measured with 10 replicates in two independent experiments. Kruskal-Wallis test with Dunn's multiple comparison test (each metabolite was compared to the no metabolite control). * = $P \leq 0.05$, ** = $P \leq 0.01$, *** = $P \leq 0.001$, **** = $P \leq 0.0001$. Data are presented as medians ± IQR. Growth of *E. faecalis* without metabolites shown in grey. Growth of *E. faecalis* with supplemented metabolites shown in shades of green. Optical density at 600 nm, OD_{600}.

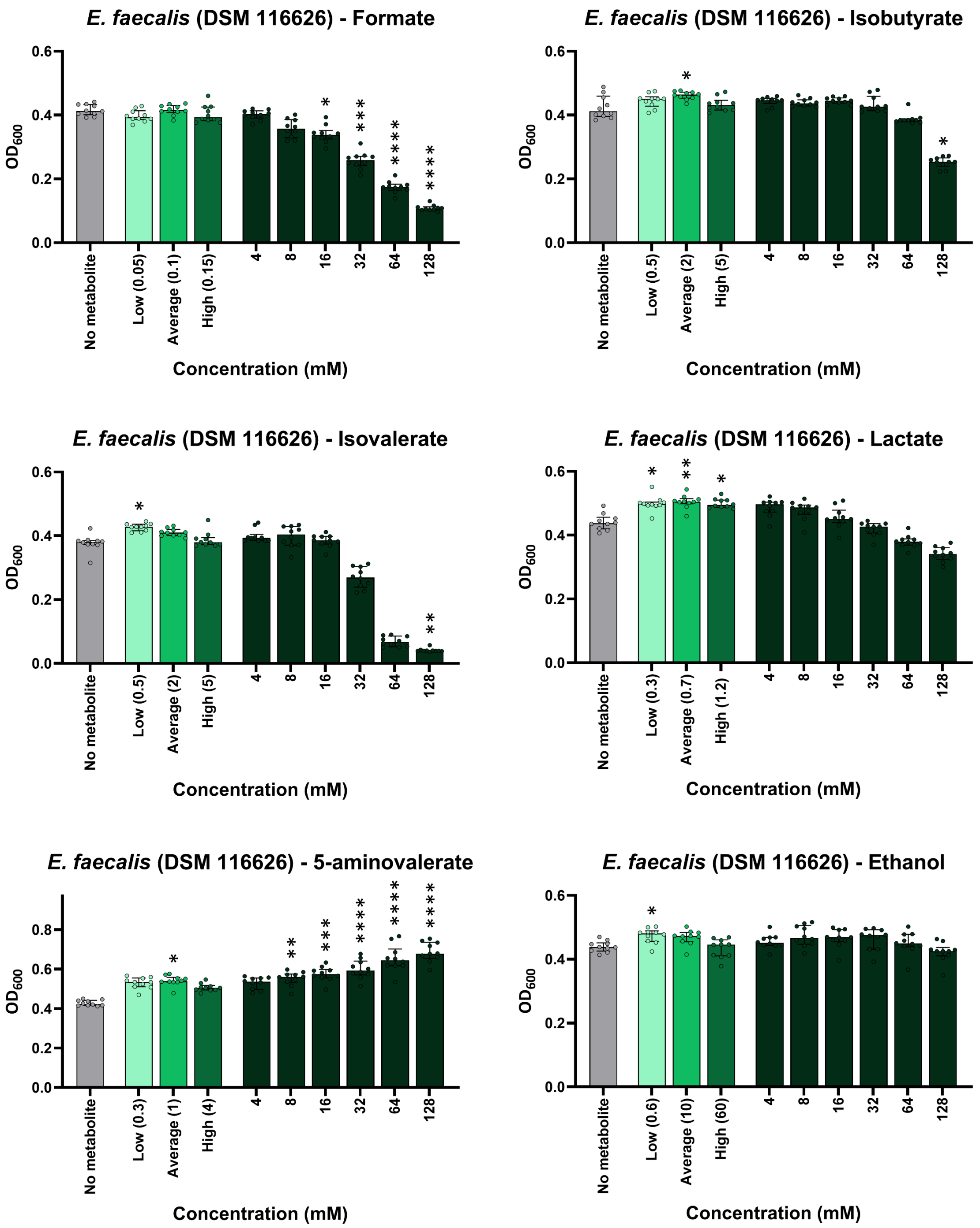

Figure S14: Some individual microbial metabolites did not suppress vancomycin-resistant *E. faecalis* (DSM 116626) growth at physiological concentrations. Vancomycin-resistant *E. faecalis* (DSM 116626) was grown in tryptic soy broth (pH 6) supplemented with an individual metabolite at low, average or high concentrations measured in healthy human faeces, concentrations spanning a defined range (4-128 mM), or unsupplemented (no metabolite control). Cultures were incubated under anaerobic conditions overnight. Growth was measured with 10 replicates in two independent experiments. Kruskal-Wallis test with Dunn's multiple comparison test (each metabolite was compared to the no metabolite control). * = $P \leq 0.05$, ** = $P \leq 0.01$, *** = $P \leq 0.001$, **** = $P \leq 0.0001$. Data are presented as medians ± IQR. Growth of *E. faecalis* without metabolites shown in grey. Growth of *E. faecalis* with supplemented metabolites shown in shades of green. Optical density at 600 nm, OD_{600}.

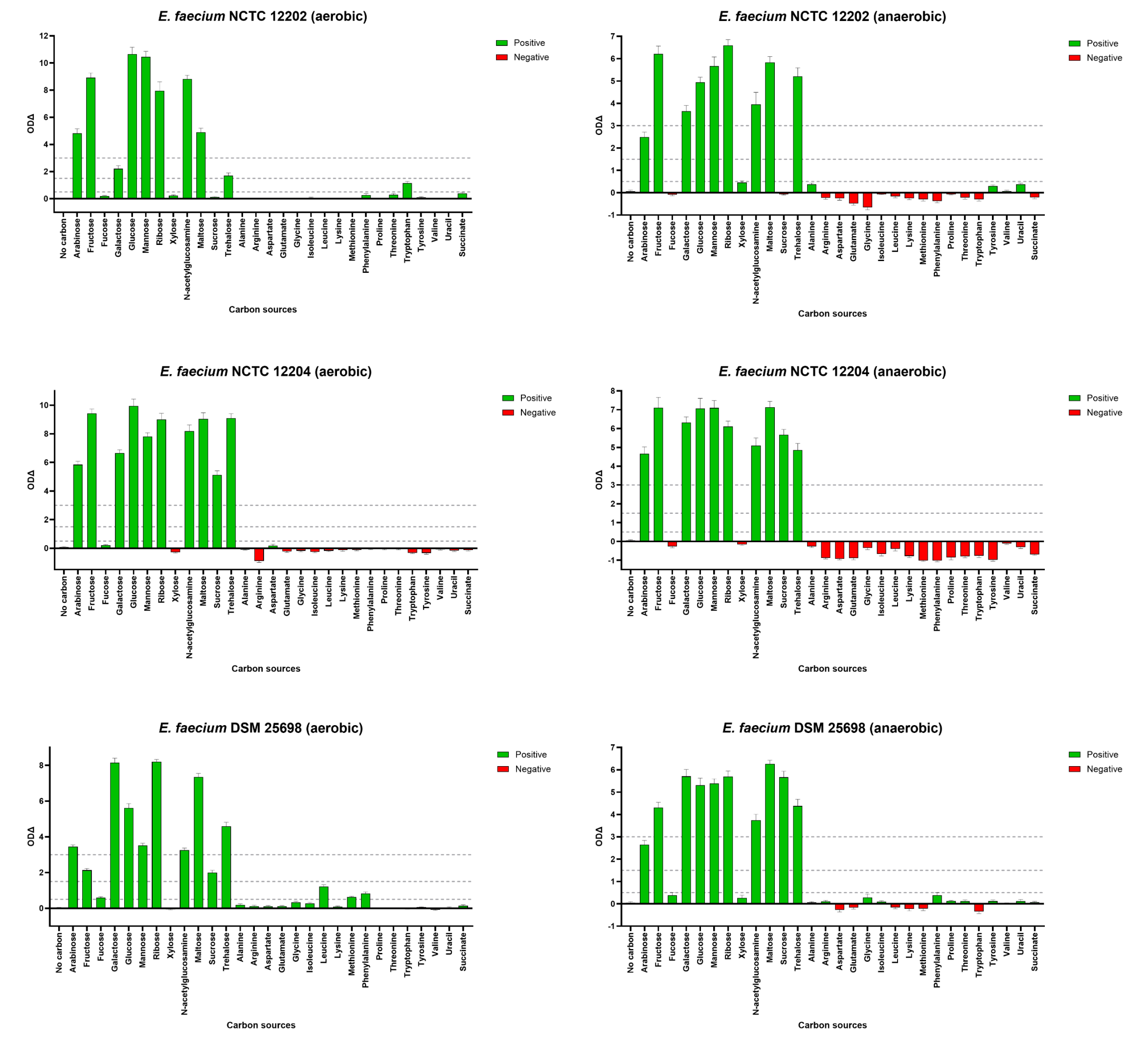

Figure S15: Vancomycin-resistant *E. faecium* growth was supported by individual nutrients found in antibiotic-treated faecal microbiomes as carbon sources. Functional differences (ODΔ) between growth of vancomycin-resistant *E. faecium* on individual carbon sources under both aerobic and anaerobic conditions. ODΔ shows the sum of functional differences between the individual carbon source growth curve compared to growth on the negative (no carbon) control. High growth is indicated by an ODΔ > 3, moderate growth is indicated by an ODΔ between 1.5 and 3, low growth is indicated by an ODΔ between 0.5 and 1.5, and negligible growth or no growth is indicated by an ODΔ < 0.5. n = 6 replicates in 2-3 independent experiments. The error bars represent the 95% confidence intervals.

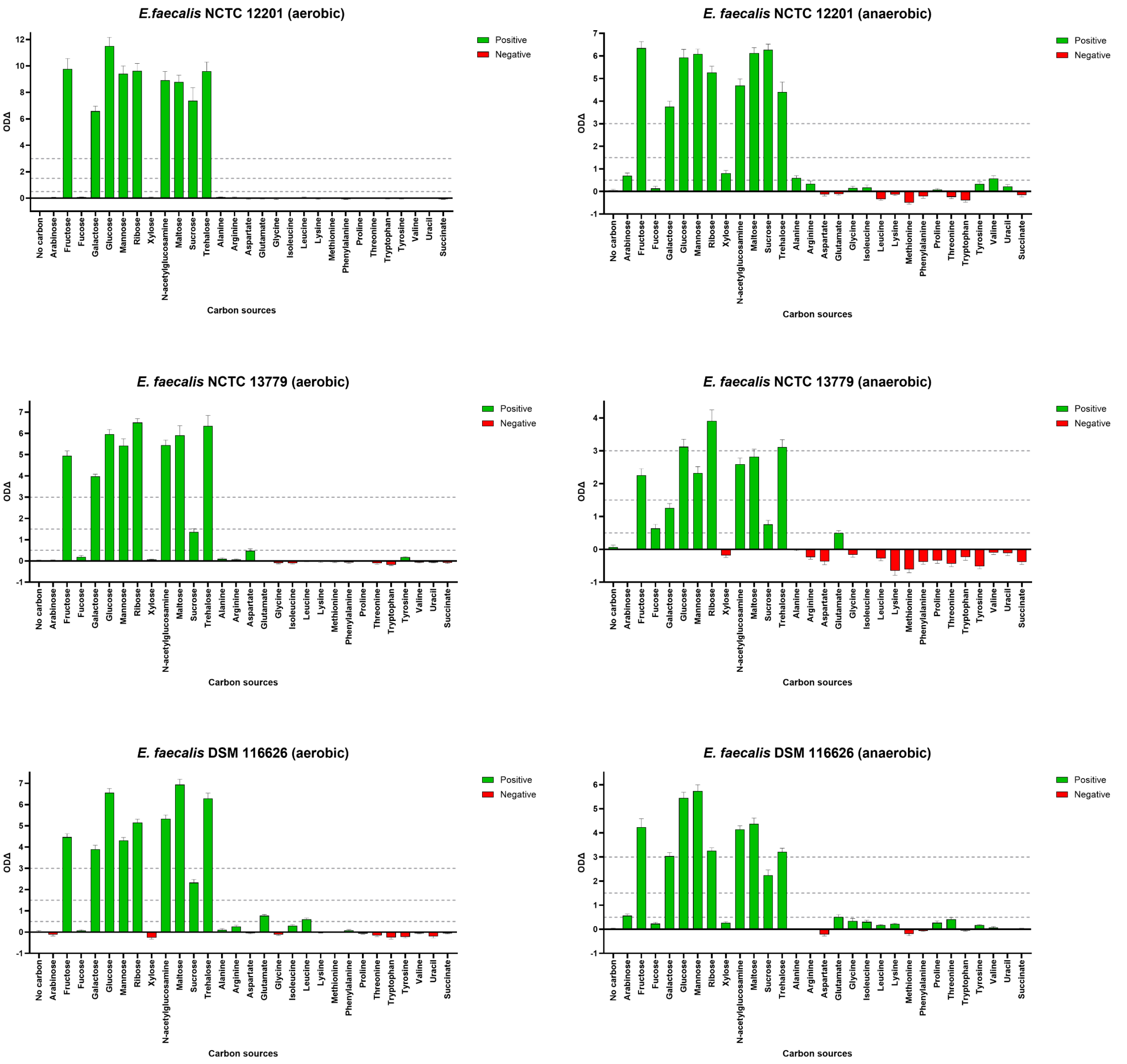

Figure S16: Vancomycin-resistant *E. faecalis* growth was supported by individual nutrients found in antibiotic-treated faecal microbiomes as carbon sources. Functional differences (ODΔ) between growth of vancomycin-resistant *E. faecalis* on individual carbon sources under both aerobic and anaerobic conditions. ODΔ shows the sum of functional differences between the individual carbon source growth curve compared to growth on the negative (no carbon) control. High growth is indicated by an ODΔ > 3, moderate growth is indicated by an ODΔ between 1.5 and 3, low growth is indicated by an ODΔ between 0.5 and 1.5, and negligible growth or no growth is indicated by an ODΔ < 0.5. n = 6 replicates in 2-3 independent experiments. The error bars represent the 95% confidence intervals.

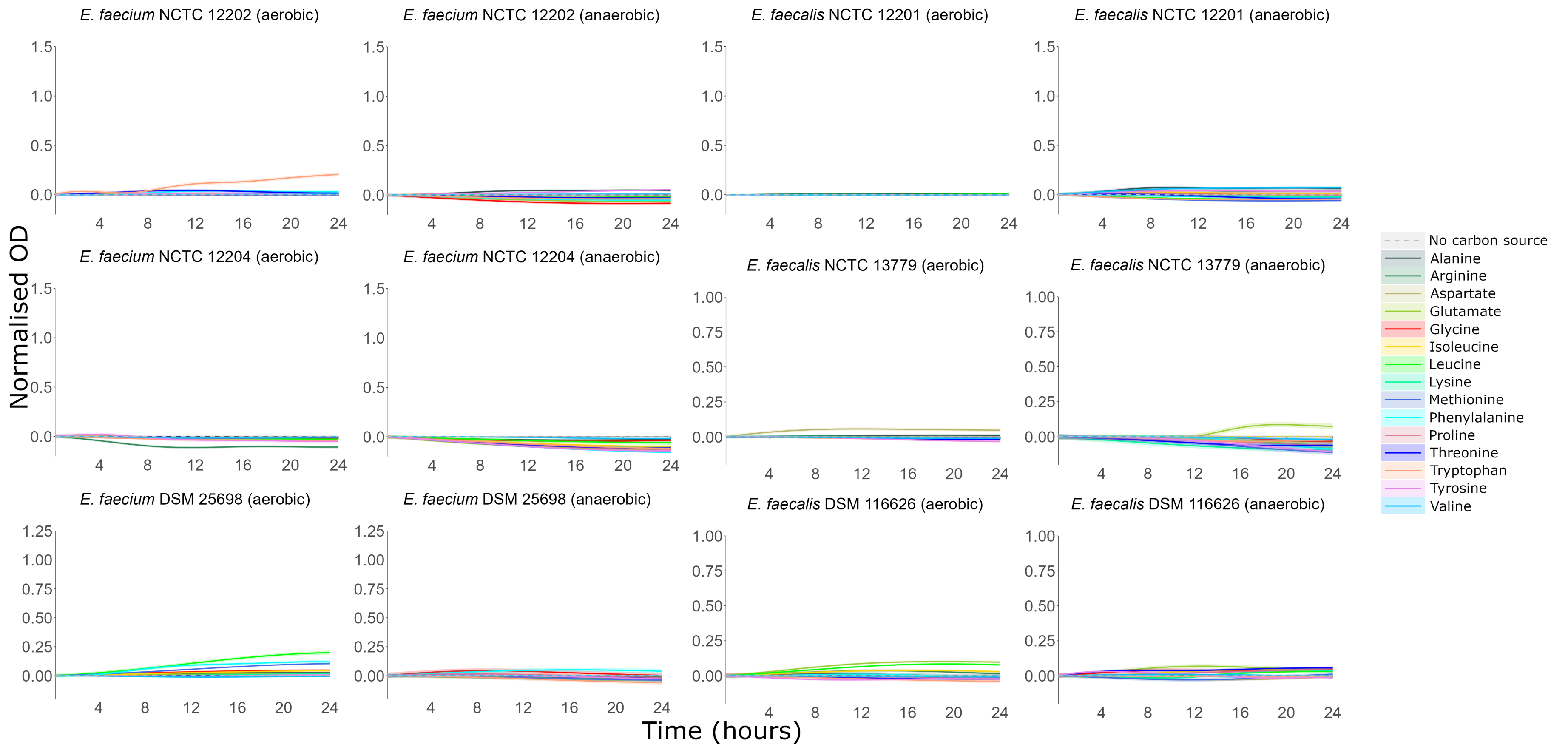

Figure S17: Amino acids supported low levels of VRE growth or did not support VRE growth as carbon sources. AMiGA-predicted growth curves for vancomycin-resistant *E. faecium* and vancomycin-resistant *E. faecalis* strains cultured in minimal medium supplemented with single carbon sources under anaerobic or aerobic conditions. The bold lines show the predicted mean of growth while the shaded bands show the predicted 95% credible intervals. n = 6 replicates in 2–3 independent experiments. OD = optical density.

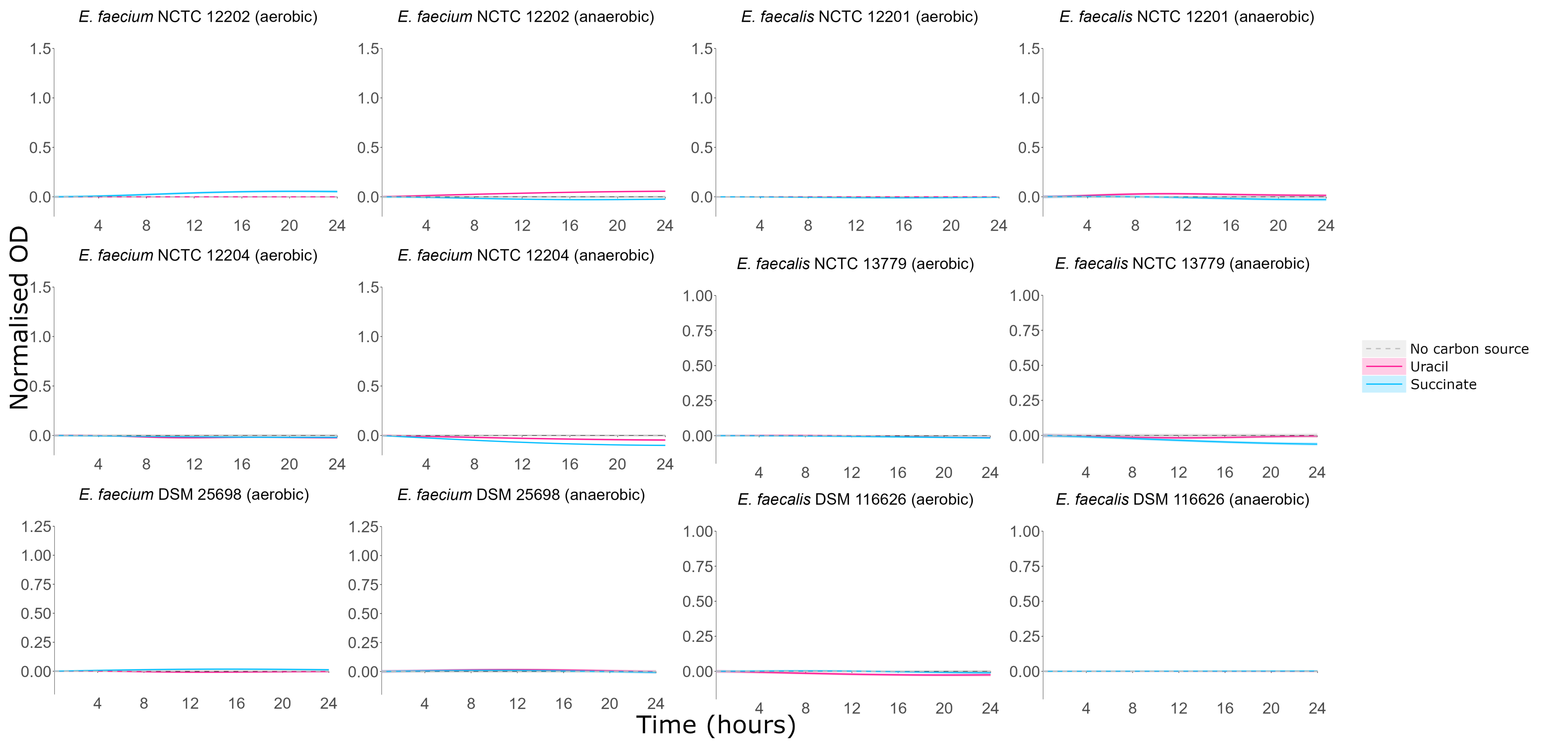

Figure S18: Uracil and succinate did not support VRE growth as carbon sources. AMiGA-predicted growth curves for vancomycin-resistant *E. faecium* and vancomycin-resistant *E. faecalis* strains cultured in minimal medium supplemented with single carbon sources under anaerobic or aerobic conditions. The bold lines show the predicted mean of growth while the shaded bands show the predicted 95% credible intervals. n = 6 replicates in 2–3 independent experiments. OD = optical density.

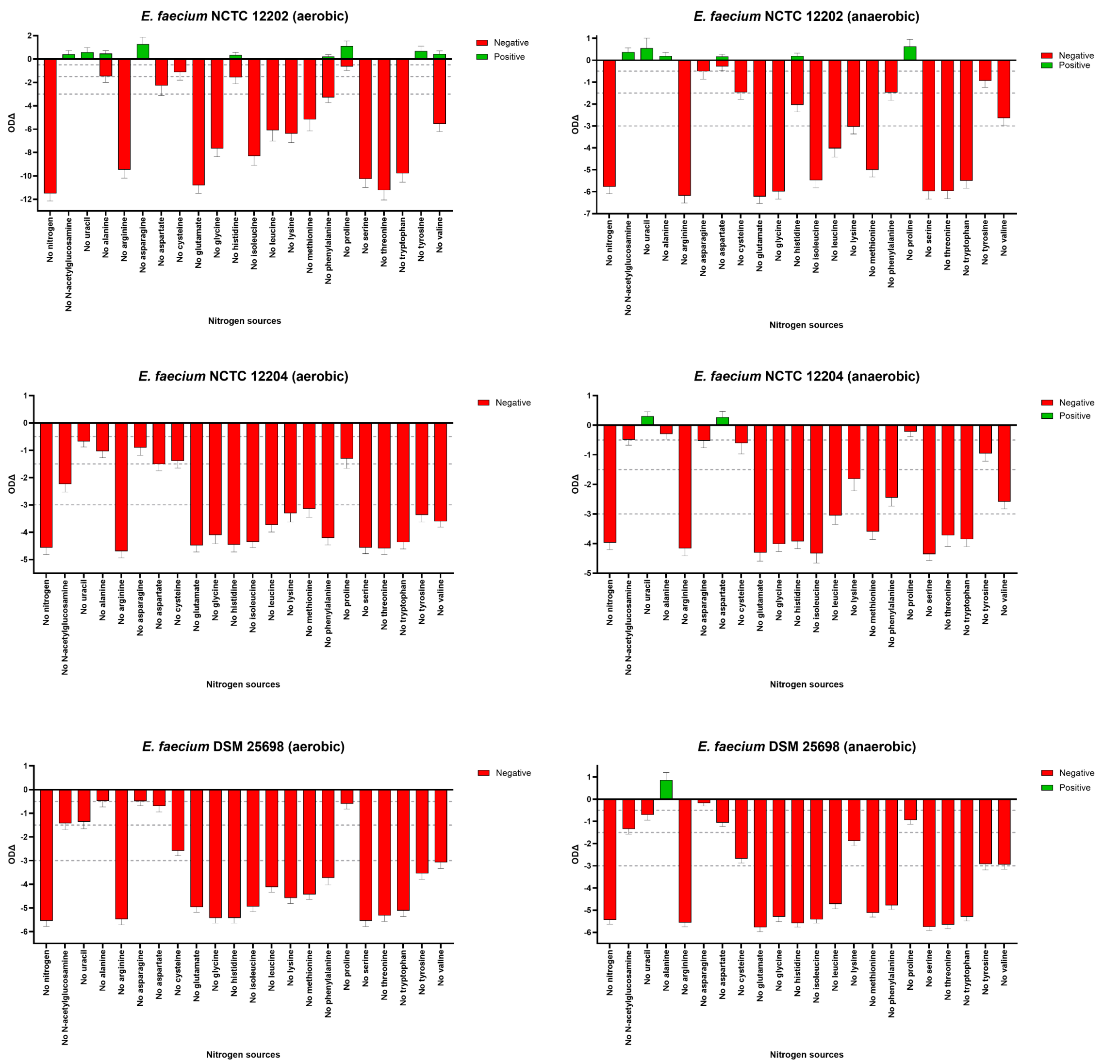

Figure S19: Vancomycin-resistant *E. faecium* growth was supported by individual nutrients found in antibiotic-treated faecal microbiomes as nitrogen sources. Functional differences (ODΔ) between growth of vancomycin-resistant *E. faecium* on nitrogen sources in a leave-one-out experiment under both aerobic and anaerobic conditions. ODΔ shows the sum of functional differences between the growth curve for each leave-one-out mixture (lacking a single nitrogen source) compared to the growth curve for the full mixture of nitrogen sources. A high decrease in growth is indicated by an ODΔ < -3, a moderate decrease in growth is indicated by an ODΔ between -1.5 and -3, a low decrease in growth is indicated by an ODΔ between -0.5 and -1.5, and negligible or no decrease in growth is indicated by an ODΔ > -0.5. n = 6 replicates in 2-3 independent experiments. The error bars represent the 95% confidence intervals.

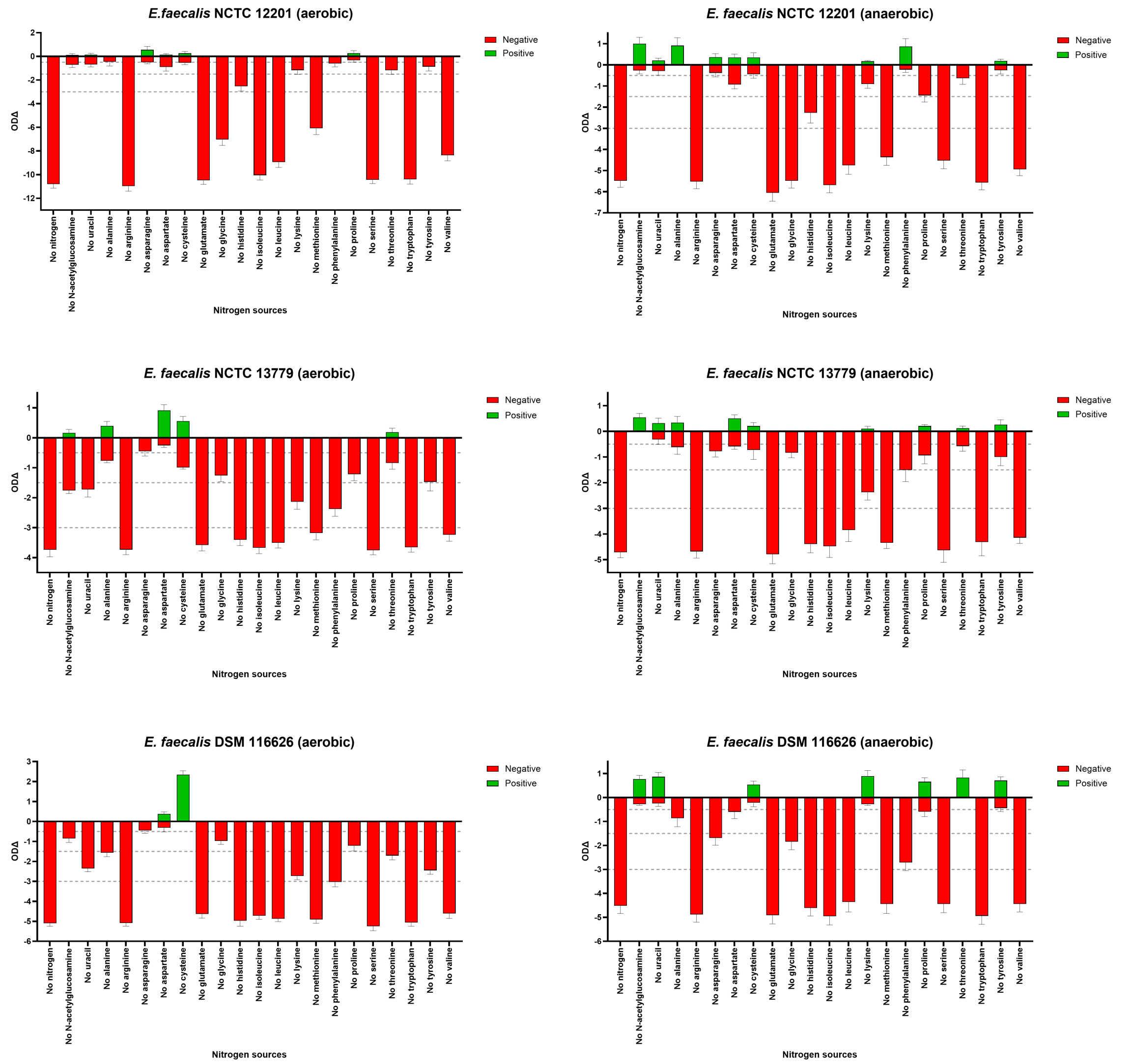

Figure S20: Vancomycin-resistant *E. faecalis* growth was supported by individual nutrients found in antibiotic-treated faecal microbiomes as nitrogen sources. Functional differences (ODΔ) between growth of vancomycin-resistant *E. faecalis* on nitrogen sources in a leave-one-out experiment under both aerobic and anaerobic conditions. ODΔ shows the sum of functional differences between the growth curve for each leave-one-out mixture (lacking a single nitrogen source) compared to the growth curve for the full mixture of nitrogen sources. A high decrease in growth is indicated by an ODΔ < -3, a moderate decrease in growth is indicated by an ODΔ between -1.5 and -3, a low decrease in growth is indicated by an ODΔ between -0.5 and -1.5, and negligible or no decrease in growth is indicated by an ODΔ > -0.5. n = 6 replicates in 2-3 independent experiments. The error bars represent the 95% confidence intervals.

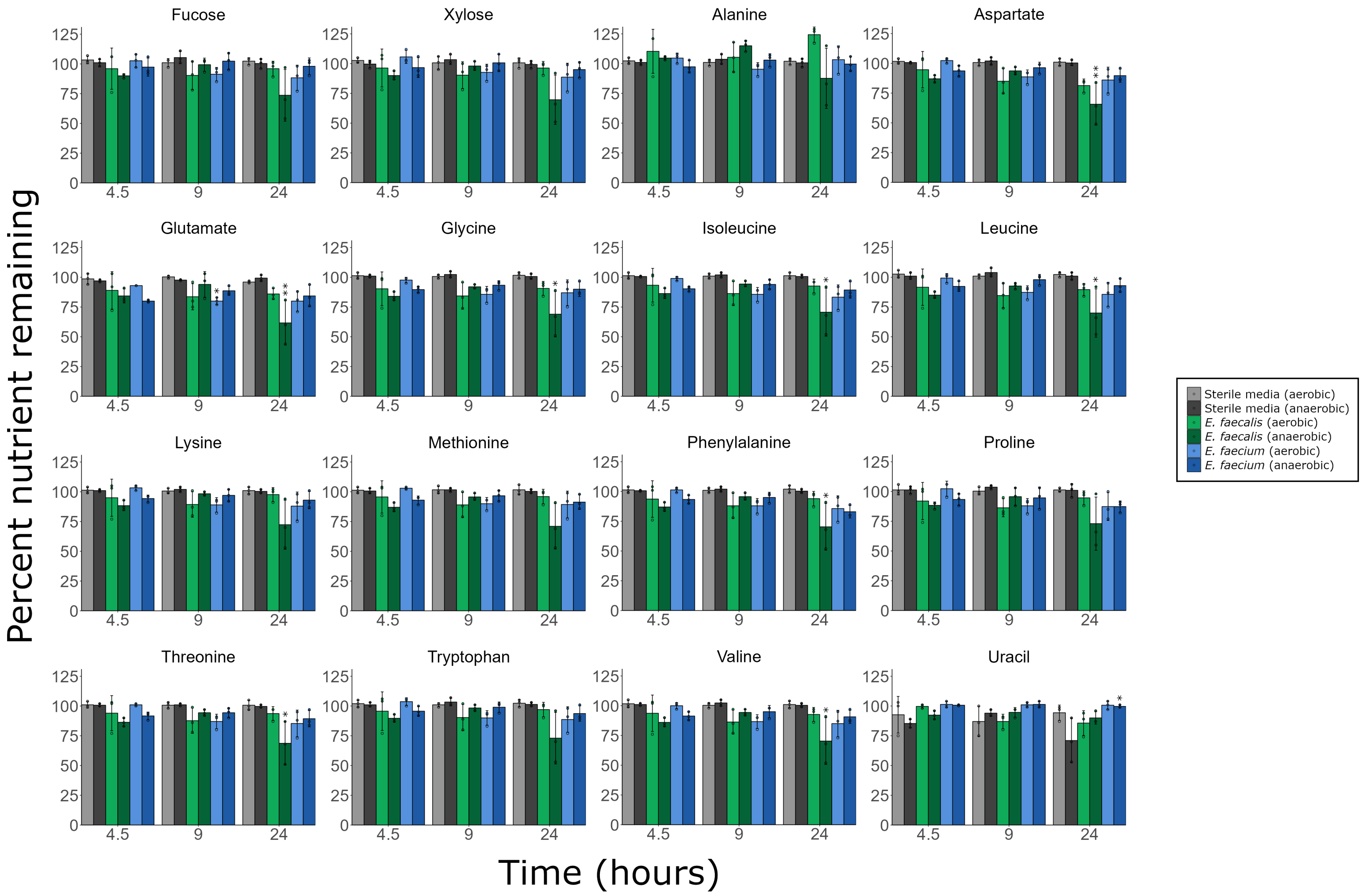

Figure S21: VRE did not prefer some nutrients over others when provided with a mixture of nutrients. Percentage of nutrients remaining in vancomycin-resistant *E. faecium* (NCTC 12202) and *E. faecalis* (NCTC 12201) cultures grown in a minimal medium supplemented with a mixture of nutrients, under aerobic or anaerobic conditions. ^{1}H-NMR spectroscopy peak integrations were used to measure nutrient concentrations. Comparisons were made using a two-way mixed ANOVA followed by pairwise comparisons with Bonferroni correction. n = 3 replicates per group, with data shown as mean ± SD. * = P ≤ 0.05, ** = P ≤ 0.01.

REFERENCES

1. Yip, A. Y. *et al*. Antibiotics promote intestinal growth of carbapenem-resistant *Enterobacteriaceae* by enriching nutrients and depleting microbial metabolites. *Nat Commun* **14**, 5094 (2023).
