# Supplementary material for: Vancomycin-resistant enterococci utilise antibiotic-enriched nutrients for intestinal colonisation
Source: Nat Commun. 2025 Jul 10;16:6376. doi: 10.1038/s41467-025-61731-z (PMC12246219; doi:10.1038/s41467-025-61731-z)
Supplement: Supplementary file 2 — Description of Additional Supplementary Files [file 41467_2025_61731_MOESM2_ESM.docx]

Description of Additional Supplementary Files

**File Name:** Supplementary Data 1

**Description:** Recipe for basal minimal medium.
