# Supplementary material for: Vancomycin-resistant enterococci utilise antibiotic-enriched nutrients for intestinal colonisation
Source: Nat Commun. 2025 Jul 10;16:6376. doi: 10.1038/s41467-025-61731-z (PMC12246219; doi:10.1038/s41467-025-61731-z)
Supplement: Supplementary file 4 — Reporting Summary [file 41467_2025_61731_MOESM4_ESM.pdf]

nature portfolio

Corresponding author(s): Julie A. K. McDonald

Last updated by author(s): Jun 23, 2025

Reporting Summary

Nature Portfolio wishes to improve the reproducibility of the work that we publish. This form provides structure for consistency and transparency in reporting. For further information on Nature Portfolio policies, see our Editorial Policies and the Editorial Policy Checklist.

Statistics

For all statistical analyses, confirm that the following items are present in the figure legend, table legend, main text, or Methods section.

n/a	Confirmed	
☐	☒	The exact sample size (*n*) for each experimental group/condition, given as a discrete number and unit of measurement
☐	☒	A statement on whether measurements were taken from distinct samples or whether the same sample was measured repeatedly
☐	☒	The statistical test(s) used AND whether they are one- or two-sided<br>*Only common tests should be described solely by name; describe more complex techniques in the Methods section.*
☐	☒	A description of all covariates tested
☐	☒	A description of any assumptions or corrections, such as tests of normality and adjustment for multiple comparisons
☐	☒	A full description of the statistical parameters including central tendency (e.g. means) or other basic estimates (e.g. regression coefficient) AND variation (e.g. standard deviation) or associated estimates of uncertainty (e.g. confidence intervals)
☐	☒	For null hypothesis testing, the test statistic (e.g. *F*, *t*, *r*) with confidence intervals, effect sizes, degrees of freedom and *P* value noted<br>*Give P values as exact values whenever suitable.*
☒	☐	For Bayesian analysis, information on the choice of priors and Markov chain Monte Carlo settings
☒	☐	For hierarchical and complex designs, identification of the appropriate level for tests and full reporting of outcomes
☒	☐	Estimates of effect sizes (e.g. Cohen's *d*, Pearson's *r*), indicating how they were calculated

Our web collection on statistics for biologists contains articles on many of the points above.

Software and code

Policy information about availability of computer code

Data collection	16S rRNA gene sequencing data was collected using the MiSeq Control Software. 16S rRNA gene qPCR data was collected using the Applied Biosystems StepOnePlus Software, NMR spectra were collected using Bruker's IconNMR component within TopSpin.
Data analysis	Statistical tests were performed using GraphPad Prism 10.1.0 (La Jolla, California) or in R (v4.2.3).<br><br>16S rRNA gene sequencing data were imported into R and processed using the standard DADA2 pipeline (version 1.18.0). 16S rRNA gene sequencing data (weighted as absolute abundances) were analysed using Wilcoxon signed rank test with Benjamini & Hochberg FDR correction using the DA.wil function within in the DAtest R package version 2.7.18.<br><br>NMR spectra were processed in Topspin (v3.2.6), imported into MATLAB r2019b, and processed using a custom script available in the Figshare repository at https://doi.org/10.6084/m9.figshare.28001687.v1 using the code available at the Zenodo repository at https://doi.org/10.5281/zenodo.3077413. Peak integration values from the 1H-NMR data were analysed using Wilcoxon signed rank test with Benjamini & Hochberg FDR correction using the DA.wil function within in the DAtest R package version 2.7.18 or unpaired t-test (two-sided) with Benjamini Hochberg FDR using the t_test function within the rstatix R package version 0.7.2. Chenomx NMR Suite v9.02 (Chenomx, Canada) was used for peak identification and representative peaks were selected and integrated.<br><br>For the mixed nutrient assays, carbon assays, and nitrogen assays the growth curves were analysed in Python (v3.6.5 or v3.12.9) using the AMiGA software (available at https://github.com/firasmidani/amiga, PMID: 34254821).

For manuscripts utilizing custom algorithms or software that are central to the research but not yet described in published literature, software must be made available to editors and reviewers. We strongly encourage code deposition in a community repository (e.g. GitHub). See the Nature Portfolio guidelines for submitting code & software for further information.

Data

Policy information about availability of data

All manuscripts must include a data availability statement. This statement should provide the following information, where applicable:

- Accession codes, unique identifiers, or web links for publicly available datasets
- A description of any restrictions on data availability
- For clinical datasets or third party data, please ensure that the statement adheres to our policy

We have provided source data with this paper. The 16S rRNA gene sequencing, 16S rRNA gene qPCR, and 1H-NMR spectroscopy datasets were deposited into the Figshare repository at https://doi.org/10.6084/m9.figshare.28001687.v1. The 16S rRNA gene sequencing raw reads have also been deposited into the ENA under BioProject PRJEB83283 (https://www.ebi.ac.uk/ena/browser/view/PRJEB83283) with sample accession numbers and links listed in the Fig. 1 Source Data file. The SILVA bacterial database version 138.1 can be found at https://www.arb-silva.de/. The compound database for the Chenomx NMR Suite v9.02 can be found at https://www.chenomx.com/.

Research involving human participants, their data, or biological material

Policy information about studies with human participants or human data. See also policy information about sex, gender (identity/presentation), and sexual orientation and race, ethnicity and racism.

Reporting on sex and gender	Male and female participants were used equally for the study as there is no biological reason to favour one sex over the other. Findings apply to both sexes and both sexes were included in this study. Sex was determined based on self-reporting. In the first faecal culture experiment, 5 males and 7 females provided faecal donations to seed the faecal cultures. In the second faecal culture experiment, 3 males and 5 females provided faecal donations to seed the faecal cultures. For the faecal culture supernatant experiment, 1 male and 2 females provided faecal donations to seed the faecal cultures.
Reporting on race, ethnicity, or other socially relevant groupings	This study did not use information on race, ethnicity, or other socially relevant groupings.
Population characteristics	Donors were between 18-65 years old and had not received antibiotic treatment in the 6+ months prior to donation.
Recruitment	Healthy donors were approached and asked if they would be interested in participating in this study. Informed consent was obtained from participants with information provided in the form of an Information Sheet detailing the research objectives and the samples needed. Samples were collected after the participant had agreed to participate in the study, had read the Information Sheet, and had signed the Informed Consent form.<br><br>As healthy donors were approached and asked to participate in our study (rather than through open advertisement to recruit donors), this study was not affected by self-selection bias. There is nothing in the donor recruitment process that would favour a particular sex, ethnicity, or other characteristic over others and participation was entirely voluntary.
Ethics oversight	Ethical approval was received by the South Central - Oxford C Research Ethics Committee (16/SC/0021 and 20/SC/0389) to collect fresh faecal samples from healthy human donors.

Note that full information on the approval of the study protocol must also be provided in the manuscript.

Field-specific reporting

Please select the one below that is the best fit for your research. If you are not sure, read the appropriate sections before making your selection.

☒ Life sciences ☐ Behavioural & social sciences ☐ Ecological, evolutionary & environmental sciences

For a reference copy of the document with all sections, see nature.com/documents/nr-reporting-summary-flat.pdf

Life sciences study design

All studies must disclose on these points even when the disclosure is negative.

Sample size	Power calculations were used to determine sample size using the appropriate statistical test (e.g. paired or unpaired t-test), an alpha of 0.05, a power of 0.80, and an effect size calculated from an appropriate existing data set.
Data exclusions	No samples were excluded from this study.
Replication	Experiments were repeated the number of times indicated in the figure legends. All attempts at replication were successful.
Randomization	Mice were randomly allocated into different groups.<br><br>For the human faecal culture experiments, each donor faecal sample was homogenised and split to seed the antibiotic-naïve group and each antibiotic-treated group. This means that each donor was allocated into all of the treatment groups.

Blinding	Investigators were not blinded for in vitro assays, ex vivo faecal culture experiments, or 1H-NMR sample preparation because the same investigator was doing group allocation during data collection. Blinding was not used for animal experiments because the investigators needed to know the treatment groups in order to administer the interventions. 16S rRNA gene sequencing library preparation was blinded.

Reporting for specific materials, systems and methods

We require information from authors about some types of materials, experimental systems and methods used in many studies. Here, indicate whether each material, system or method listed is relevant to your study. If you are not sure if a list item applies to your research, read the appropriate section before selecting a response.

Materials & experimental systems

n/a	Involved in the study
☒	☐ Antibodies
☒	☐ Eukaryotic cell lines
☒	☐ Palaeontology and archaeology
☐	☒ Animals and other organisms
☒	☐ Clinical data
☒	☐ Dual use research of concern
☒	☐ Plants

Methods

n/a	Involved in the study
☒	☐ ChIP-seq
☒	☐ Flow cytometry
☒	☐ MRI-based neuroimaging

Animals and other research organisms

Policy information about studies involving animals; ARRIVE guidelines recommended for reporting animal research, and Sex and Gender in Research

Laboratory animals	Female wild-type C57BL/6 mice (8-10 weeks old) were purchased from Envigo (Huntingdon, UK). Mice were housed 5 per cage in individually ventilated cages with bedding (Aspen chip 2 bedding, NEPCO, Warrensburg, New York). Mice were maintained at 20-22°C and 45-65% humidity, with 12-hour light and dark cycles. Mice were given autoclaved food (RM1, Special Diet Services, Essex, UK) and water (provided ad libitum). Prior to the start of the experiment mice were acclimatised for 1 week.
Wild animals	N/A
Reporting on sex	Female C57BL/6 mice were used in this study. The findings of this study apply to both sexes.
Field-collected samples	N/A
Ethics oversight	The Imperial College London Animal Welfare and Ethical Review Body (PF93C158E) provided ethical approval for the mouse experiments.

Note that full information on the approval of the study protocol must also be provided in the manuscript.

Plants

Seed stocks	*Report on the source of all seed stocks or other plant material used. If applicable, state the seed stock centre and catalogue number. If plant specimens were collected from the field, describe the collection location, date and sampling procedures.*
Novel plant genotypes	*Describe the methods by which all novel plant genotypes were produced. This includes those generated by transgenic approaches, gene editing, chemical/radiation-based mutagenesis and hybridization. For transgenic lines, describe the transformation method, the number of independent lines analyzed and the generation upon which experiments were performed. For gene-edited lines, describe the editor used, the endogenous sequence targeted for editing, the targeting guide RNA sequence (if applicable) and how the editor was applied.*
Authentication	*Describe any authentication procedures for each seed stock used or novel genotype generated. Describe any experiments used to assess the effect of a mutation and, where applicable, how potential secondary effects (e.g. second site T-DNA insertions, mosiacism, off-target gene editing) were examined.*
